# *Arctium* Species Secondary Metabolites Chemodiversity and Bioactivities

**DOI:** 10.3389/fpls.2019.00834

**Published:** 2019-07-09

**Authors:** Dongdong Wang, Alexandru Sabin Bădărau, Mallappa Kumara Swamy, Subrata Shaw, Filippo Maggi, Luiz Everson da Silva, Víctor López, Andy Wai Kan Yeung, Andrei Mocan, Atanas G. Atanasov

**Affiliations:** ^1^The Second Affiliated Hospital of Guizhou University of Traditional Chinese Medicine, Guiyang, China; ^2^Department of Molecular Biology, Institute of Genetics and Animal Breeding of the Polish Academy of Sciences, Jastrzębiec, Poland; ^3^Department of Pharmacognosy, Faculty of Life Sciences, University of Vienna, Vienna, Austria; ^4^Department of Environmental Science, Faculty of Environmental Science and Engineering, Babeş-Bolyai University, Cluj-Napoca, Romania; ^5^Department of Biotechnology, East West First Grade College of Science, Bengaluru, India; ^6^Center for the Development of Therapeutics, Broad Institute of MIT and Harvard, Cambridge, MA, United States; ^7^School of Pharmacy, University of Camerino, Camerino, Italy; ^8^Postgraduate Program in Sustainable Territorial Development, Federal University of Paraná, Curitiba, Brazil; ^9^Department of Pharmacy, Faculty of Health Sciences, Universidad San Jorge, Villanueva de Gállego, Spain; ^10^Instituto Agroalimentario de Aragón-IA2, CITA-Universidad de Zaragoza, Zaragoza, Spain; ^11^Oral and Maxillofacial Radiology, Applied Oral Sciences, Faculty of Dentistry, The University of Hong Kong, Hong Kong, China; ^12^Department of Pharmaceutical Botany, Faculty of Pharmacy, “Iuliu Haţieganu” University of Medicine and Pharmacy, Cluj-Napoca, Romania; ^13^Laboratory of Chromatography, Institute of Advanced Horticulture Research of Transylvania, University of Agricultural Sciences and Veterinary Medicine, Cluj-Napoca, Romania; ^14^Institute of Neurobiology, Bulgarian Academy of Sciences, Sofia, Bulgaria

**Keywords:** *Arctium* species, secondary metabolites, volatile compounds, non-volatile compounds, chemodiversity, bioactivity

## Abstract

*Arctium* species are known for a variety of pharmacological effects due to their diverse volatile and non-volatile secondary metabolites. Representatives of *Arctium* species contain non-volatile compounds including lignans, fatty acids, acetylenic compounds, phytosterols, polysaccharides, caffeoylquinic acid derivatives, flavonoids, terpenes/terpenoids and volatile compounds such as hydrocarbons, aldehydes, methoxypyrazines, carboxylic and fatty acids, monoterpenes and sesquiterpenes. *Arctium* species also possess bioactive properties such as anti-cancer, anti-diabetic, anti-oxidant, hepatoprotective, gastroprotective, antibacterial, antiviral, antimicrobial, anti-allergic, and anti-inflammatory effects. This review aims to provide a complete overview of the chemistry and biological activities of the secondary metabolites found in therapeutically used *Arctium* species. Summary of pharmacopeias and monographs contents indicating the relevant phytochemicals and therapeutic effects are also discussed, along with possible safety considerations.

## Introduction

### Botanical and Ethnobotanical Aspects

The genus *Arctium* L. (Asteraceae/Compositae, tribe Cardueae, subtribe Carduinae), together with the related genera *Cousinia* Cass., *Hypacanthium* Juz. and *Schmalhausenia* C. Winkl, forms the so-called *Arctium–Cousinia* group (de Souza et al., 2004). The species of the *Arctium* genus, also known as ‘burdock,’ comprise biennial herbs occurring in waste places, streams and roadsides, less often in wood and forests, in temperate regions of Europe and Asia and sporadically in subtropical and tropical regions (European Scientific Cooperative on Phytotherapy, 2003). In North and South America, the genus is considered as naturalized, whereas in Africa it is quite rare. The name of the genus comes from the Greek ‘*arcteion*’ which means ‘bear,’ alluding to the plant habitus characterized by pronounced hairiness.

According to the Plant List^1^, this genus encompasses 18 recognized species among which five are considered as hybrid species due to the frequent outbreeding occurring between its allogamous representatives (Lopez-Vinyallonga et al., 2010).

*Arctium* species are represented by hemicryptophyte plants equipped with a stout, erect taproot and entire (sporadically as dentate), rough, unarmed, alternate, tomentose, and cordate leaves. The stem is usually stout, erect, grooved, branched, and reddish. Inflorescences are formed by solitary or corymbose ovoid-conical to spherical capitula equipped with involucres made up of bracts ending with hooked apices. Receptacles are composed of numerous, hard scales. Florets are only tubulose, hermaphrodite, purple or white. Pollination is allowed by insects, mostly belonging to Lepidoptera. Fruits are having oblong, rugose achenes equipped with a golden-yellow pappus (European Scientific Cooperative on Phytotherapy, 2003).

**FIGURE 1:**
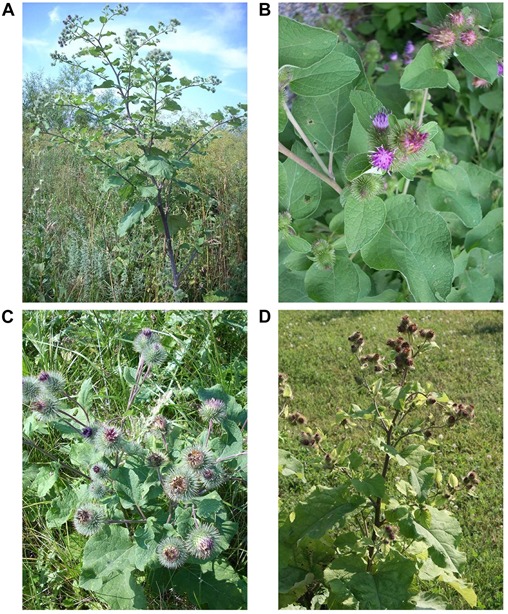
Plants of *Arctium*. **(A)** *Arctium lappa*; **(B)** *Arctium minus*; **(C)** *Arctium tomentosum*; **(D)** *Arctium nemorosum*.

The *Arctium* genus is highly polymorphic due to variability occurring in hairiness of leaves and capitula, length of floral peduncles, and color of capitula and florets. As a consequence, a sharp distinction between its members cannot occasionally be defined. In the Euro-Mediterranean area, six main species are found: *A. atlanticum* (Pomel) H. Lindb., *A. lappa* L., *A. minus* (Hill) Bernh., *A. nemorosum* Lej., *A. palladini* (Marcow) R.E.Fr. & Soderb. and *Arctium tomentosum* Mill. (European Scientific Cooperative on Phytotherapy, 2003; Figure 1).

*Arctium lappa* enjoys a longstanding use in the traditional medicine (mainly roots, and, to a lesser extent, leaves and seeds) due to the bioactive properties of its metabolites (Zhao et al., 2014). To the best of our knowledge, the most utilized species for therapeutic purposes, are on first place *A. lappa*, also known as ‘greater burdock,’ and, to a minor extent, *A. minus* (lesser burdock) and *A. tomentosum* (wooly burdock).

*Arctium lappa* is an herbaceous biennial plant up to 150 cm tall, with pubescent to subglabrous epigeal parts. Basal leaves are up to 50 cm in diameter, ovate, cordate and with hollow petioles. Stems are branched and end with corymbose capitula. Florets are as long as the involucral bracts. *A. minus* differs for the shape of inflorescence (solitary terminal capitula), dimensions of involucral bracts and capitula (smaller and shorter, respectively) and consistency of petiole (hollow). Furthermore, the bracts show a relatively hinted hairiness. *A. tomentosum* is characterized by petioles and peduncles covered with wooly tufts; petioles are solid. The involucral bracts are similar in dimensions to those of *A. minus*, but they show a dense covering of hairs. Florets are longer than bracts as in *A. minus* (European Scientific Cooperative on Phytotherapy, 2003). These three species are quite common in central Europe where often they undergo interspecific hybridization giving rise to questions about their integrity (European Scientific Cooperative on Phytotherapy, 2003).

Greater burdock (*A. lappa*) has been traditionally used in both Asian and European medicines as depurative, diuretic, carminative, anti-inflammatory, and anti-tubercular agent (Zhao et al., 2014). For therapeutic purposes, its different parts such as roots, fruits, and leaves are used. The latter have been used to treat ulcers and fester wounds (Jaric et al., 2007). They are also applied externally on the forehead to cure headache and fever, on the scalp to treat bruises and hair loss, mixed with oil and honey and applied on the chest to heal cough. In addition, under infusion, they are taken orally to treat enuresis in children (Pieroni et al., 2011). Fruits of burdock (*Arctii fructus*) are used to purify the blood (Lans and Turner, 2011) and to treat respiratory and infectious diseases (Bai et al., 2016). In addition, *A. lappa* roots, together with aerial parts of *Rumex acetosella* L., leaves of *Ulmus rubra* Muhl. and rhizomes of *Rheum officinale* Baill., are used to make ‘Essiac,’ a tea used by the Ojibwa tribe of Canada for the treatment of cancer (Leonard et al., 2006). In the veterinary medicine, the root is used to treat mastitis (Lans et al., 2007), whereas the whole plant is applied against endoparasites in poultry (Lans and Turner, 2011). Besides therapeutic uses, *A. lappa* is also appreciated as an edible plant. For the latter purpose, young leaves, and stalks are eaten raw or cooked (Pieroni et al., 2011).

Lesser burdock (*A. minus*) leaves are traditionally used externally to treat rheumatic pains, fever, sunstroke, wounds, general infections, skin and body inflammations, alopecia, and bladder diseases (de Souza et al., 2004; Erdemoglu et al., 2009; Neves et al., 2009). They are also disposed above the body of the patient, wetted with vinegar or milk, to stimulate sweating (Sezik et al., 2001). Roots and leaves, under infusion, are also used against snake and scorpion bites and to purify the blood (Mosaddegh et al., 2012). Basal leaves and stems are also eaten raw as a snack or stewed (Tardio et al., 2005). Due to their bitter taste, they are also used to stimulate the appetite and liver functions (Tardio et al., 2005).

Wooly burdock (*A. tomentosum*) leaves are used as vulnerary, to treat skin rash, ulcers, abscesses, mouth sores and against rheumatic pains, whereas root is applied against alopecia and to wash hairs (Sezik et al., 2004; Saric-Kundalic et al., 2010). Roots are also employed to make a tea used for digestive problems, ulcers, rheumatisms, to purify the blood and increase sweating and as diuretic (Saric-Kundalic et al., 2010).

### Medicinal Uses of *Arctium* Species in Pharmacopeias and Monographs

Burdock species and in particular *A. lappa* are used in traditional medicine for different purposes. The main traditional use of the roots of *A. lappa* in Europe comprises treatment of dermatological disorders (Saric-Kundalic et al., 2010; Miglani and Manchanda, 2014) whereas in other Eastern and Asian countries *A. lappa* fruits and roots are used as an antidiabetic remedy (Tousch et al., 2014; Xu et al., 2014, 2015; Ahangarpour et al., 2017). In Traditional Chinese Medicine (TCM), apart from the antidiabetic activity, the roots of *A. lappa* are considered as a blood detoxifying agent (Qin et al., 2014). In the Japanese pharmacopeia, the fruit is included as a traditional herbal medicine with recent studies revealing the potential of its extracts in oncology (Ikeda et al., 2016). The leaves of *A. lappa* have also been reported as an anti-inflammatory agent to relieve gastrointestinal disorders in Brazilian traditional medicine (de Almeida et al., 2013).

According to international institutions that work in the validation of traditional herbal medicines, such as the European Medicines Agency (EMA) and the European Scientific Cooperative on Phytotherapy (ESCOP), *A. lappa* is recommended and approved for different indications. For example, the EMA monograph approves the use of roots of *A. lappa, A. minus*, and *A. tomentosum* as an adjuvant in minor urinary tract complaints, in temporary loss of appetite and for seborrheic skin conditions (European Medicines Agency, 2011). All these indications are based upon long-standing use. In 2016, ESCOP released a monograph where the roots of all the former three species are indicated to be internally and externally used for seborrheic skin, eczema, furuncles, acne, psoriasis and internally for minor urinary tract disorders (European Scientific Cooperative on Phytotherapy-The Scientific Foundation for Herbal Medicinal Products, 2016). For oral and internal administration, the herbal drug can be used as an infusion, extract, tincture or decoction but the fresh pulp of the roots or a decoction can also be directly applied to the skin. The later monograph reveals that *A. lappa* preparations should not be ingested during pregnancy, lactation, or in case of hypersensitivity to the Compositae and in patients with oedema due to impaired heart or kidney function. Although certain preclinical studies can be found in the literature, clinical trials are not available for these indications approved by ESCOP and EMA.

## Phytochemistry

### Non-volatile Compounds

**FIGURE 2:**
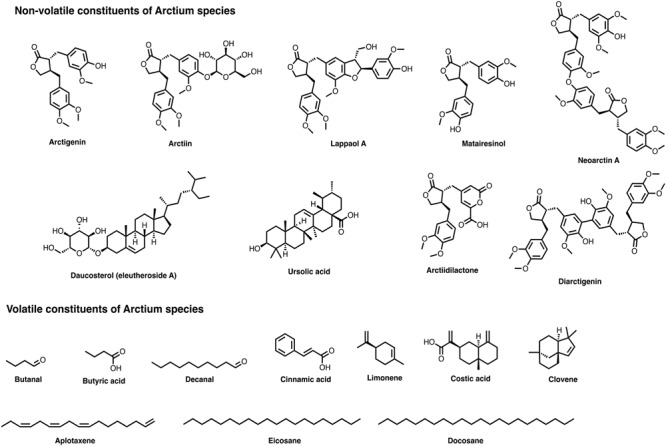
Chemical structures of several relevant components present in *Arctium* species.

Till date, more than two hundred non-volatile compounds have been isolated from *Arctium* genus. These chemical compounds include lignans, terpenoids, sterols, flavonoids, phenolics, lactones, polyacetylenes, quinic acids, and sugars (polysaccharides). In particular, lignans are the most characteristic components in the *Arctium* genus. The details of chemical compounds, their occurrence in different plant parts, and the analytical methods used for their quali-quantitative determinations are briefly summarized in Table 1 whereas their description is provided in this section. The chemical structures of some compounds from *Arctium* species are shown in Figure 2.

**Table 1:** The known non-volatile constituents of *Arctium* species.

No	Compound name	Formula	Species	Plant origin/part	Analytical method	References
	Lignans					
1	Diarctigenin	C_42_H_46_O_12_	*A. lappa*	Fruits, roots, seeds	IR/NMR/MS/TLC	Han et al., 1994; Park et al., 2007; Qin et al., 2014
2	Arctiin	C_27_H_34_O_11_	*A. lappa, A. tomentosum*	Leaves, fruits, roots, seeds	UV/IR/MS/NMR/HPLC/LCMS/ MALDI-QIT-TOF MS	Wang and Yang, 1993; Ting-Guo et al., 2001; Yu et al., 2003; Ming et al., 2004; Liu et al., 2005, 2012, 2015; Wang et al., 2005; Matsumoto et al., 2006; Boldizsar et al., 2010; Ferracane et al., 2010; Zhou et al., 2011; Qin et al., 2014; Su et al., 2015; Lou et al., 2016; Al-Shammaa et al., 2017
3	Arctigenin	C_12_H_24_O_7_	*A. lappa, A. tomentosum*	Leaves, fruits, seeds, roots	UV/MS/NMR/HPLC/LCMS/ MALDI-QIT-TOF MS/ HRESI-MS	Umehara et al., 1993; Wang and Yang, 1993; Liu et al., 2005, 2012, 2015; Matsumoto et al., 2006; Gao et al., 2008; Boldizsar et al., 2010; Ferracane et al., 2010; Predes et al., 2011; Zhou et al., 2011; Qin et al., 2014; Su et al., 2015; Al-Shammaa et al., 2017
4	Arctigenin-4-*O*-β-D-gentiobioside	C_18_ H_32_ *O*_16_	*A. lappa*	Fruits	NMR/UV/IR/ORD/HRESIMS	Yang et al., 2015
5	Arctigenin-4-*O*-α-D-galactopyranosyl-(1→6)-*O*-β-D-glucopyranoside	C_18_ H_32_ *O*_16_	*A. lappa*	Fruits	NMR/UV/IR/ORD/HRESIMS	Yang et al., 2015
6	Arctigenin-4-*O*-β-D-apiofuranosyl-(1→6)-*O*-β-D-glucopyranoside	C_32_ H_42_ O_15_	*A. lappa*	Fruits	NMR/UV/IR/ORD/HRESIMS	Yang et al., 2015
7	3-benzyl-6-(1-hydroxyethyl)-2,5-piperazinedione	C_13_ H_16_ N*_2_*O_3_	*A. lappa*	Fruits	IR/HR-ESI-MS/NMR/CD	Yang et al., 2012
8	3-benzyl-2,5- piperazinedione	C_13_H_16_N*_2_*O_2_	*A. lappa*	Fruits	IR/HR-ESI-MS/NMR/CD	Yang et al., 2012
9	5′-propanediolmatairesinoside	C_29_ H_38_O_13_	*A. lappa*	Fruits	NMR/UV/IR/ORD/HRESIMS	Yang et al., 2015
10	(7′R,8R,8’R)-rafanotrachelogenin-4-*O*-β-D-glucopyranoside	C_27_ H_34_ O_12_	*A. lappa*	Fruits	NMR/UV/IR/ORD/HRESIMS	Yang et al., 2015
11	(7′S,8R,8′R)-rafanotrachelogenin-4-*O*-β-D-glucopyranoside	C_27_ H_34_ O_12_	*A. lappa*	Fruits	NMR/UV/IR/ORD/HRESIMS	Yang et al., 2015
12	(7S,8S,8’R)-4,7-dihydroxy-3,3’,4-trimethoxyl-9-oxo benzylbutyrolactone lignan-4-*O*-β-D-glucopyranoside	C_27_ H_34_O_12_	*A. lappa*	Fruits	NMR/UV/IR/ORD/HRESIMS	Yang et al., 2015
13	(7S,8S,8’R)-4,7- dihydroxy-3,3′,4′-trimethoxyl-9-oxo dibenzylbutyrolactone lignin	C_21_H_24_O_7_	*A. lappa*	Fruits	NMR/UV/IR/ORD/HRESIMS	Yang et al., 2015
14	(7R,8S,8′R)-4,7,4′-trihydroxy-3,3′- dimethoxyl-9-oxo dibenzylbutyrolactone lignan-4-*O*-β-D-glucopyranoside	C_26_ H_32_O_12_	*A. lappa*	Fruits	NMR/UV/IR/ORD/HRESIMS	Yang et al., 2015
15	7,8-didehydroarctigenin	C21H22O5	*A. lappa*	Fruits	HRFAB/EIMS/NMR	Matsumoto et al., 2006
16	Arctiidilactone	C20H20O8	*A. lappa*	Fruits	NMR/UV/IR/ORD/HRESIMS	Yang et al., 2015
17	Arctiiapolignan A	C20H28O10	*A. lappa*	Fruits	NMR/UV/IR/ORD/HRESIMS	Yang et al., 2015
18	Arctiisesquineolignan A	C42H52O19	*A. lappa*	Fruits	NMR/UV/IR/ORD/HRESIMS	Yang et al., 2015
19	Arctiisesquineolignan B	C36H46O16	*A. lappa*	Fruits	UV/IR/HRESIMS/NMR	He etal., 2016
20	Arctiiphenolglycoside A	C_19_H_28_O_13_	*A. lappa*	Fruits	UV/IR/HRESIMS/NMR	He etal., 2016
21	Arctignan A	C_30_H_34_O_10_	*A. lappa*	Seeds	UV/MS/NMR/HPLC	Umehara et al., 1993
22	Arctignan B	C_30_H_34_O_10_	*A. lappa*	Seeds	UV/MS/NMR/HPLC	Umehara et al., 1993
23	Arctignan C	C_30_H_32_O_10_	*A. lappa*	Seeds	UV/MS/NMR/HPLC	Umehara et al., 1993
24	Arctignan D	C_30_H_34_O_10_	*A. lappa*	Seeds	UV/MS/NMR/HPLC/LCMS/ MALDI-QIT-TOF MS	Umehara et al., 1993; Liu et al., 2012
25	Arctignan E	C_40_H_44_O_13_	*A. lappa*	Seeds	UV/IR/MS/NMR/HPLC	Umehara et al., 1993; Ming et al., 2004; Ferracane et al., 2010; Qin et al., 2014
26	Lappaol A	C_30_H_32_O_9_	*A. lappa, A. tomentosum*	Seeds/fruits	TLC/UV/IR/MS/NMR/HPLC	Ichihara et al., 1976; Ting-Guo et al., 2001; Ming et al., 2004; Ferracane et al., 2010; Liu et al., 2012; Qin et al., 2014; Su et al., 2015
27	Lappaol B	C_31_H_34_O_9_	*A. lappa*	Seeds/fruits	NMR/MS/TLC/HPLC	Ichihara et al., 1976; Qin et al., 2014
28	Isolappaol C	C_30_H_34_O_10_	*A. lappa, A. tomentosum*	Seeds/fruits	NMR/MS/TLC	Park et al., 2007; Qin et al., 2014; Su et al., 2015
29	Lappaol C	C_30_H_34_O_10_	*A. lappa*	Seeds	TLC/UV/IR/MS/NMR	Ichihara et al., 1977; Ting-Guo et al., 2001; Ming et al., 2004; Park et al., 2007; Ferracane et al., 2010; Liu et al., 2012; Su et al., 2015
30	Lappaol D	C_31_H_36_O_10_	*A. lappa*	Seeds	NMR/MS/TLC	Ichihara et al., 1977; Park et al., 2007
31	Lappaol E	C_30_H_34_O_10_	*A. lappa*	Seeds	NMR/MS/TLC	Ichihara et al., 1977; Park et al., 2007
32	Lappaol F	C_42_H_46_O_12_	*A. lappa, A. tomentosum*	Fruits, seeds	TLC/UV/IR/MS/NMR/HPLC	Ting-Guo et al., 2001; Ming et al., 2004; Park et al., 2007; Ferracane et al., 2010; Qin et al., 2014
33	Lappaol H	C_40_H_46_O_14_	*A. lappa*	Seeds/fruits	UV/MS/NMR/HPLC/LCMS/ MALDI-QIT-TOF MS	Liu et al., 2012; Qin et al., 2014
34	Neoarctin A	C_42_H_46_O_12_	*A. lappa*	Seeds	UV, IR, 1H-NMR, 13C-NMR, DEPT, 2D-NMR and MS	Wang and Yang, 1995; Yong et al., 2007
35	Neoarctin B	C_42_H_46_O_12_	*A. lappa*	Seeds	UV, IR, 1H-NMR, 13C-NMR, DEPT, 2D-NMR and MS	Wang and Yang, 1993
36	Matairesinoside	C_26_H_32_O_11_	*A. lappa*	Fruits	UV/IR/HPLC	Boldizsar et al., 2010
37	Matairesinol	C_20_H_22_O_6_	*A. lappa*	Seeds/fruits	UV/MS/NMR/HPLC/LCMS/ MALDI-QIT-TOF MS	Wang and Yang, 1993; Boldizsar et al., 2010; Ferracane et al., 2010; Liu et al., 2012; Qin et al., 2014; Su et al., 2015
38	Matairesinol-4,4′-di-*O*-β-D-glucopyranoside	C_27_ H_34_ O_12_	*A. lappa*	Fruits	NMR/UV/IR/ORD/HRESIMS	Yang et al., 2015
39	Pinoresinol	C_20_H_22_O_6_	*A. lappa*	Fruits	UV/IR/HPLC	Boldizsar et al., 2010
40	Phylligenin	C_21_H_24_O_6_	*A. lappa*	Fruits	UV/IR/HPLC	Boldizsar et al., 2010
41	Styraxlignolide E	C_26_H_32_O_11_	*A. lappa*	Fruits	NMR/UV/IR/ORD/HRESIMS	Yang et al., 2015
42	Styraxlignolide D	C_26_H_32_O_11_	*A. lappa*	Fruits	NMR/UV/IR/ORD/HRESIMS	Yang et al., 2015
43	Syringaresinol	C_22_H_26_O_8_	*A. lappa*	Roots	UV/IR/ESIMS/NMR	Han et al., 2013
44	(7S, 8R)-4,7,9,9′-tetrahydroxy-3,3′-dimethoxyl-7′-oxo-8-4′-oxyneolignan-4-*O*-β-D-glucopyranoside	C_26_ H_34_ O_13_	*A. lappa*	Roots	IR/HR-ESI-MS/NMR/CD	Yang et al., 2012
45	(7′*S*, 8′*R*, 8*S*)-4,4′,9′-trihydroxy-3,3′-dimethoxy-7′,9-epoxylignan-7-oxo-4-*O*-β-D-glucopyranosyl-4′-*O*-β-D-glucopyranoside	C_32_ H_42_ O_17_	*A. lappa*	Roots	IR/HR-ESI-MS/NMR/CD	Yang et al., 2012
46	(7S, 8R)-4,7,9,9′-tetrahydroxy-3,3′dimethoxy-8-*O*-4′-neolignan-9′-*O*-β-D-apiofuranosyl-(1 → 6)-*O*-β-D-glucopyranoside	C_31_H_44_O_16_	*A. lappa*	Fruits	IR/HR-ESI-MS/NMR/CD	Huang K. et al., 2015; Huang X.Y. et al., 2015
47	(8R)-4,9,9′-trihydroxy-3,3′-dimethoxy-7-oxo-8-*O*-4′-neolignan-4-*O*-β-D-glucopyranoside	C_26_ H_34_ O_12_	*A. lappa*	Fruits	IR/HR-ESI-MS/NMR/CD	Huang K. et al., 2015; Huang X.Y. et al., 2015
48	(7R, 8S)-dihydrodehydrodiconiferyl alcohol-7′-oxo-4-*O*-β-D-glucopyranoside	C_26_ H_32_ O_12_	*A. lappa*	Fruits	IR/HR-ESI-MS/NMR/CD	Huang K. et al., 2015; Huang X.Y. et al., 2015
49	(7′S, 8′R, 8S)-4,4′,9′-trihydroxy-3,3′-dimethoxy-7′,9-epoxylignan-7-oxo-4-*O*-β-D-glucopyranoside	C_26_ H_32_ O_12_	*A. lappa*	Fruits	IR/HR-ESI-MS/NMR/CD	Huang K. et al., 2015; Huang X.Y. et al., 2015
50	Trachelogenin	C_21_H_24_O_7_	*A. lappa*	Fruits	–	Ichikawa et al., 1986
	Terpenes/Terpenoids					
51	β-eudesmol	C_15_H_26_O	*A. lappa*	Fruits	–	Yayli et al., 2005
52	Ursolic acid	C_30_H_48_O_3_	*A. lappa*	Root	UV/IR/ESIMS/NMR	Han et al., 2013
53	Oleanolic acid	C_30_H_48_O_3_	*A. lappa*	Roots	UV/IR/ESIMS/NMR	Han et al., 2013
54	Arctiopicrin	C_19_H_26_O_6_	*A. lappa, A. minus*	Leaves	TLC/NMR	Savina et al., 2006
55	Onopordopicrin	C_19_H_24_O_6_	*A. lappa, A. nemorosum*	Leaves/aerial parts	TLC/HPLC/NMR/HR-ESI-TOF-MS	Barbosa et al., 1993; Savina et al., 2006; Machado et al., 2012; Zimmermann et al., 2012
56	Dehydrovomifoliol	C_13_H_18_O_3_	*A. lappa*	Leaves	NMR/HR-ESI-TOF-MS	Machado et al., 2012
57	Loliolide	C_11_H_16_O_3_	*A. lappa*	Leaves	NMR/HR-ESI-TOF-MS	Machado et al., 2012
58	Dehydromelitensin-8-(4′-hydroxymethacrylate)	C_15_ H_24_ O_6_	*A. lappa*	Leaves	NMR/HR-ESI-TOF-MS	Machado et al., 2012
59	Dehydromelitensin	C_15_H_20_O_4_	*A. lappa*	Leaves	NMR/HR-ESI-TOF-MS	Machado et al., 2012
60	Melitensin	C_15_H_22_O_4_	*A. lappa*	Leaves	NMR/HR-ESI-TOF-MS	Machado et al., 2012
61	3α-acetoxyhop-22(29)-ene	C_30_H_49_O_2_	*A. lappa*	Leaves	NMR, IR and MS	Jeelani and Khuroo, 2012
62	3α-hydroxylanosta-5,15-diene	C_30_ H_50_O	*A. lappa*	Leaves	NMR, IR and MS	Jeelani and Khuroo, 2012
	Flavonoids					
63	Baicalin	C_21_H_18_O_11_	*A. lappa*			Uchiyama et al., 2005
64	Luteolin	C_25_H_24_O_12_	*A. lappa*	Leaves/roots	UPLC/LC/MS/MS	Ferracane et al., 2010; Lou et al., 2010a; Tang et al., 2014
65	Rutin	C_27_H_30_O_16_	*A. lappa, A. minus*	Leaves	TLC/UPLC/LC/MS/MS	Saleh and Bohm, 1971; Lou et al., 2010a,b
66	Quercitrin	C_21_H_20_O_11_	*A. lappa*	Leaves/roots	UPLC/LC/MS/MS	Lou et al., 2010a
67	Quercetin	C_15_H_10_O_7_	*A. lappa*	Leaves/roots	UPLC/LC/MS/MS/HRESI-MS	Lou et al., 2010a; Predes et al., 2011; Tang et al., 2014
68	Quercetin 3-*O*-glucuronide	C_21_H_18_O_13_	*A. lappa*	Roots	HPTLC/LC/ESI–MS/MS	Rajasekharan et al., 2015
69	Quercetin 3-vicianoside	C_26_H_28_O_16_	*A. lappa*	Roots	HPTLC/LC/ESI–MS/MS	Rajasekharan et al., 2015
70	Quercetin rhamnoside	C_21_H_20_O_11_	*A. lappa*	roots	HPLC/LC/MS/MS	Ferracane et al., 2010
71	Quercimeritrin	C_21_H_20_O_12_	*A. minus*	Leaves	TLC	Saleh and Bohm, 1971
72	Isoquercetin	C_21_H_20_O_12_	*A. minus*	Leaves	TLC	Saleh and Bohm, 1971
73	Astragalin	C_21_H_20_O_11_	*A. minus*	Leaves	TLC	Saleh and Bohm, 1971
74	Kaempferol-3-o-rhamnoglucoside	C_27_ H_30_ O_15_	*A. minus*	Leaves	TLC	Saleh and Bohm, 1971
75	Biachanin A	C_16_H_12_O_5_	*A. lappa*	Roots	–	Tamayo et al., 2000; Eberding et al., 2007
76	Genestein	C_15_H_10_O_5_	*A. lappa*	Roots	–	Tamayo et al., 2000; Eberding et al., 2007
77	Nobiletin	C_21_H_22_O_8_	*A. lappa*	Roots	–	Tamayo et al., 2000; Eberding et al., 2007
78	Tangeretin	C_20_H_20_O_7_	*A. lappa*	Roots	–	Tamayo et al., 2000
	Sterols					
79	β-sitosterol	C_29_H_50_O	*A. lappa, A. tomentosum*	Seeds/roots/fruits	UV/IR/MS/NMR/HPLC	Ting-Guo et al., 2001; Ming et al., 2004; Han et al., 2013
80	Sitosterol-beta-D-glucopyranoside	C_35_ H_60_ O_6_	*A. lappa*	Roots	IR/NMR/EI-MS	Miyazawa et al., 2005
81	Daucosterol	C_35_H_60_O_6_	*A. lappa, A. tomentosum*	Seeds/fruits	UV, IR, 1H-NMR, 13C-NMR, DEPT, 2D-NMR and MS/HPLC	Wang and Yang, 1993; Ting-Guo et al., 2001; Han et al., 2013
	Fatty acids					
82	Docosanoic acid	C_22_H_44_O_2_	*A. tomentosum*	Seeds	GCMS	Zong et al., 2013
83	Eicosanoic acid	C_20_H_40_O_2_	*A. tomentosum*	Seeds	GCMS	Zong et al., 2013
84	*cis*-13-eicosenoic acid	C_20_H_38_O_2_	*A. tomentosum*	Seeds	GCMS	Zong et al., 2013
85	Methyl palmitate	C_17_H_34_O_2_	*A. lappa*	–	IR/NMR/EI-MS	Miyazawa et al., 2005
86	Methyl linoleate	C_19_H_34_O_2_	*A. lappa*	–	IR/NMR/EI-MS	Miyazawa et al., 2005
87	Methyl linolenate	C_19_H_32_O_2_	*A. lappa*	Roots	IR/NMR/EI-MS/GCMS	Miyazawa et al., 2005; Kuo et al., 2012
88	Methyl stearate	C_19_H_38_O_2_	*A. lappa*	–	IR/NMR/EI-MS	Miyazawa et al., 2005
89	Methyl oleate	C_19_H_36_O_2_	*A. lappa*	Roots	IR/NMR/EI-MS/GCMS	Miyazawa et al., 2005; Kuo et al., 2012
90	Hexadecanoic acid	C_16_H_32_O_2_	*A. lappa, A. tomentosum*	Fruits/seeds	UV/TLC/IR/NMR/EIMS/GCMS	Miyazawa et al., 2005; Boldizsar et al., 2010; Zong et al., 2013
91	9-hexadecenoic acid	C_16_H_30_O_2_	*A. tomentosum*	Seeds	GCMS	Zong et al., 2013
92	Linoleic acid	C_18_H_32_O_2_	*A. lappa*	Roots	IR/NMR/EI-MS/GCMS	Miyazawa et al., 2005; Boldizsar et al., 2010; Kuo et al., 2012
93	Linolenic acid	C_18_H_30_O_2_	*A. lappa*	Fruits	IR/NMR/EI-MS; GCMS	Miyazawa et al., 2005
94	Stearic acid	C_17_H_35_CO_2_H	*A. lappa*	Fruits	IR/NMR/EI-MS	Miyazawa et al., 2005
95	9,12-octadecadienoic acid	C_18_H_32_O_2_	*A. tomentosum*	Seeds	GCMS	Zong et al., 2013
96	Oleic acid	C_18_H_34_O_2_	*A. lappa*	Fruits	UV/IR/HPLC/NMR/EI-MS	Miyazawa et al., 2005; Boldizsar et al., 2010
97	Oxiraneoctanoic acid	C_19_H_36_O_3_	*A. tomentosum*	Seeds	GCMS	Zong et al., 2013
98	Tetracosanoic acid	C_24_H_48_O_2_	*A. tomentosum*	Seeds	GCMS	Zong et al., 2013
	Acetylenic compounds					
99	Arctinone-a	C_13_H_10_O_2_S_2_	*A. lappa*	Roots	UV/TLC/IR/NMR/MS	Washino et al., 1986
100	Arctinone-b	C_13_H_10_OS_2_	*A. lappa*	Roots	UV/TLC/IR/NMR/MS	Washino et al., 1986
101	Arctinol-a	C_13_H_12_O_2_S_2_	*A. lappa*	Roots	UV/TLC/IR/NMR/MS	Washino et al., 1986
102	Arctinol-b	C_13_H_12_O_2_S_2_	*A. lappa*	Roots	UV/TLC/IR/NMR/MS	Washino et al., 1986
103	Arctinal	C_12_H_8_OS_2_	*A. lappa*	Roots	UV/TLC/IR/NMR/MS	Washino et al., 1986
104	Arctic acid-b	C_13_H_8_O_3_S_2_	*A. lappa*	Roots	UV/TLC/IR/NMR/MS	Washino et al., 1986
105	Arctic acid-c	C_13_H_10_O_3_S_2_	*A. lappa*	Roots	UV/TLC/IR/NMR/MS	Washino et al., 1986
106	Methyl arctate-b	C_14_H_10_O_3_S_2_	*A. lappa*	Roots	UV/TLC/IR/NMR/MS	Washino et al., 1986
107	Arctinone-a acetate	C_15_H_10_O_3_S_2_	*A. lappa*	Roots	UV/TLC/IR/NMR/MS	Washino et al., 1986
108	Dehydrodihydrocostus lactone	C_15_H_21_O_2_	*A. lappa*	Roots	UV/TLC/IR/NMR/MS	Washino et al., 1986, 1987
109	Dehydrocostus lactone	C_15_H_19_O_2_	*A. lappa*	Roots	UV/TLC/IR/NMR/MS	Washino et al., 1986, 1987
110	Lappaphen-a	C_27_H_26_O_4_S	*A. lappa*	Roots	UV/TLC/IR/NMR/MS	Washino et al., 1986, 1987
111	Lappaphen-b	C_27_H_26_O_4_S	*A. lappa*	Roots	UV/TLC/IR/NMR/MS	Washino et al., 1986, 1987
	Carboxylic acids/Quinic acids and derivatives					
112	Caffeic acid	C_9_H_8_O_4_	*A. lappa*	Seeds/leaves/roots	TLC/HPLC/UPLC/LC/MS/ HRESI-MS	Chen et al., 2004; Lin and Harnly, 2008; Lou et al., 2010a,b; Ferracane et al., 2010; Predes et al., 2011; Tang et al., 2014; Lou et al., 2016; Al-Shammaa et al., 2017
113	Caffeic acid 4-*O*-glucoside	C_15_H_18_O_9_	*A. lappa*	Roots	LC-DAD-ESI/MS	Lin and Harnly, 2008; Lou et al., 2016
114	Chlorogenic acid	C_16_H_18_O_9_	*A. lappa*	Seeds/leaves/roots	TLC/HPTLC/HPLC/UPLC/LC/MS/MALDI-QIT-TOF MS/ HRESI-MS	Wang et al., 2001; Chen et al., 2004; Lin and Harnly, 2008; Ferracane et al., 2010; Lou et al., 2010a,b; Predes et al., 2011; Liu et al., 2012; Haghi et al., 2013; Qin et al., 2014; Liu et al., 2015; Lou et al., 2016; Al-Shammaa et al., 2017
115	*p*-coumaric acid	C_9_H_8_O_3_	*A. lappa*	Seeds/leaves/roots	UPLC/EIMS	Lou et al., 2010a,b; Tang et al., 2014
116	Coumaroylquinic acid	C_16_H_18_O_8_	*A. lappa*	Roots	HPTLC/LC/ESI–MS/MS	Rajasekharan et al., 2015
117	Benzoic Acid	C_7_H_6_O_2_	*A. lappa*	Leaves	UPLC/EIMS	Lou et al., 2010a,b
118	Cynarin	C_25_H_24_O_12_	*A. lappa*	Seeds/leaves/roots	UPLC/LC/MS	Ferracane et al., 2010; Lou et al., 2010a, 2016; Tang et al., 2014
119	Caffeoyl-hexose-hydroxyphenol	C_21_ H_21_O_10_	*A. lappa*	Roots	HPTLC/LC/ESI–MS/MS	Rajasekharan et al., 2015
120	1-*O*-caffeoylquinic acid	C_16_H_18_O_9_	*A. lappa*	Roots	GCMS/LC-DAD-ESI/MS	Lin and Harnly, 2008; Tousch et al., 2014
121	3-*O*-caffeoylquinic acid	C_16_H_18_O_9_	*A. lappa*	Roots	GCMS/LC-DAD-ESI/MS	Lin and Harnly, 2008; Tousch et al., 2014
122	4-*O*-caffeoylquinic acid	C_16_H_18_O_9_	*A. lappa*	Roots	GCMS/LC-DAD-ESI/MS	Lin and Harnly, 2008; Tousch et al., 2014
123	5-*O*-caffeoylquinic acid	C_16_H_18_O_9_	*A. lappa*	Roots	GCMS/LC-DAD-ESI/MS	Lin and Harnly, 2008; Jaiswal and Kuhnert, 2011; Tousch et al., 2014
124	1-*O*-,5-*O*-dicaffeoylquinic acid	C_25_H_24_O_12_	*A. lappa*	Roots	HPLC/NMR/MS	Maruta et al., 1995; Wang et al., 2001; Han et al., 2013; Rajasekharan et al., 2015
125	1-*O*-, 5-*O*-dicaffeoyl-3-*O*-succinylquinaiccid	C_35_ H_40_ 0_15_	*A. lappa*	Roots	NMR/EI-MS	Maruta et al., 1995
126	1-*O*,-5-*O*-dicaffeoyl-4-*O*-succinylquinic acid	C_29_H_35_0_15_	*A. lappa*	Roots	NMR/MS	Maruta et al., 1995
127	1-*O*-,5-O-dicaffeoyl-3-*O*-	C_33_H_39_0_18_	*A. lappa*	Roots	NMR/MS	Maruta et al., 1995
128	4-*O*-disuccinylquaicniidc and 1-*O*-,3-*0*-,5-*O*-tricaffeoyl-4-*O*-succinylquinic acid	C_38_H_41_0_18_	*A. lappa*	Roots	NMR/MS	Maruta et al., 1995
129	1,3-di-*O*-caffeoylquinic acid	C_25_H_24_O_12_	*A. lappa*	Seeds/roots	LCMS/ MALDI-QIT-TOF MS	Lin and Harnly, 2008; Liu et al., 2012
130	1,5-di-*O*-caffeoylquinic acid	C_25_H_24_O_12_	*A. lappa*	Leaves/Seeds/roots	UPLC/HPLC/PDA/LCMS/ MALDI-QIT-TOF MS	Maruta et al., 1995; Lin and Harnly, 2008; Liu et al., 2012; Haghi et al., 2013; Tousch et al., 2014
131	1,5-di-*O*-caffeoyl-4-*O*-maloylquinic acid	C_29_H_27_O_16_	*A. lappa*	Roots	LCMS/ MALDI-QIT-TOF MS	Jaiswal and Kuhnert, 2011; Liu et al., 2012; Tousch et al., 2014
132	1,5-di-*O*-caffeoyl-3-*O*-maloylquinic acid	C_25_H_27_O_16_	*A. lappa*	Roots	LCMS/ MALDI-QIT-TOF MS	Jaiswal and Kuhnert, 2011; Liu et al., 2012; Tousch et al., 2014
133	1,5-di-*O*-caffeoyl-3-*O*-succinoylquinic acid	C_29_H_27_O_15_	*A. lappa*	Roots	LCMS/ MALDI-QIT-TOF MS	Maruta et al., 1995; Jaiswal and Kuhnert, 2011; Liu et al., 2012; Tousch et al., 2014
134	1,5-di-*O*-caffeoyl-3,4-di-*O*-succinoylquinic acid	C_33_H_31_O_18_	*A. lappa*	Roots	LCMS/ MALDI-QIT-TOF MS	Liu et al., 2012; Tousch et al., 2014
135	1,3,5-tri-*O*-caffeoyl-4-*O*-succinoylquinic acid	C_38_H_33_O_18_	*A. lappa*	Roots	GCMS/LCMS/ MALDI-QIT-TOF MS	Jaiswal and Kuhnert, 2011; Liu et al., 2012; Tousch et al., 2014
136	1,3,5-tri-*O*-caffeoylquinic acid	C_34_H_29_O_15_	*A. lappa*	Roots	GCMS/LCMS/ MALDI-QIT-TOF MS	Liu et al., 2012; Tousch et al., 2014
137	1,5-di-*O*-caffeoyl-3-*O*-succinoyl-4-*O*-maloyquinic acid	–	*A. lappa*	Roots	LCMS/ MALDI-QIT-TOF MS	Liu et al., 2012
138	5-sinapoylquinic acid	C_18_H_22_O_10_	*A. lappa*	Roots	LC-DAD-ESI/MS	Lin and Harnly, 2008
139	3-sinapoyl-5-caffeoylquinic acid	C_27_H_28_O_13_	*A. lappa*	Roots	LC-DAD-ESI/MS	Lin and Harnly, 2008
140	3-sinapoyl-5-caffeoyl-1-methoxyoxaloylquinic acid	–	*A. lappa*	Roots	LC-DAD-ESI/MS	Lin and Harnly, 2008
141	4-sinapoyl-5-caffeoyl-1-methoxyoxaloylquinic acid	–	*A. lappa*	Roots	LC-DAD-ESI/MS	Lin and Harnly, 2008
142	3,4-dicaffeoylquinic acid	C_25_H_24_O_12_	*A. lappa*	Roots/seeds	LC-DAD-ESI/MS	Lin and Harnly, 2008
143	1,4-di-*O*-caffeoylquinic acid	C_25_H_23_O_12_	*A. lappa*	Roots	GCMS/LC-DAD-ESI/MS	Lin and Harnly, 2008; Jaiswal and Kuhnert, 2011; Tousch et al., 2014
144	3,5-di-*O*-caffeoylquinic acid	C_25_H_24_O_12_	*A. lappa*	Roots	GCMS/LC-DAD-ESI/MS	Lin and Harnly, 2008; Jaiswal and Kuhnert, 2011; Tousch et al., 2014
145	4,5-dicaffeoylquinic acid	C_25_H_24_O_12_	*A. lappa*	Roots/seeds	LC-DAD-ESI/MS	Lin and Harnly, 2008
146	3,5-dicaffeoyl-1-methoxyoxaloylquinic acid	–	*A. lappa*	Roots	LC-DAD-ESI/MS	Lin and Harnly, 2008
147	3-feruloyl-5-caffeoylquinic acid	–	*A. lappa*	Roots	LC-DAD-ESI/MS	Lin and Harnly, 2008
148	4,5-dicaffeoyl-1-methoxyoxaloylquinic acid	–	*A. lappa*	Roots	LC-DAD-ESI/MS	Lin and Harnly, 2008
149	3-sinapoyl-5-caffeoyl-4-methoxyoxaloylquinic acid	–	*A. lappa*	Roots	LC-DAD-ESI/MS	Lin and Harnly, 2008
150	1,4,5-tricaffeoylquinic acid	C_34_H_30_O_15_	*A. lappa*	Roots	LC-DAD-ESI/MS	Lin and Harnly, 2008
151	3,4,5-tricaffeoylquinic acid	–	*A. lappa*	Roots	LC-DAD-ESI/MS	Lin and Harnly, 2008
152	1,4,5-tricaffeoyl-3-methoxyoxaloylquinic acid	–	*A. lappa*	Roots	LC-DAD-ESI/MS	Lin and Harnly, 2008
153	3-succinoyl-4,5-dicaffeoyl	–	*A. lappa*	Roots	LCMS	Jaiswal and Kuhnert, 2011
154	1,5-dicaffeoyl-3-succinoylquinic acid	–	*A. lappa*	Roots	HPLC/LCMS	Wang et al., 2001; Jaiswal and Kuhnert, 2011
155	1,5-di-*O*-caffeoyl-4-*O*-succinoylquinic acid	C_29_H_27_O_15_	*A. lappa*	Roots	GCMS/LCMS	Maruta et al., 1995; Jaiswal and Kuhnert, 2011; Liu et al., 2012; Tousch et al., 2014
156	3,4-dicaffeoyl-5-succinoylquinic acid	C_29_H_28_O_15_	*A. lappa*	Roots	LCMS	Jaiswal and Kuhnert, 2011
157	1,3-dicaffeoyl-5-fumaroylquinic acid	–	*A. lappa*	Roots	LCMS	Jaiswal and Kuhnert, 2011
158	1,5-dicaffeoyl-4-fumaroylquinic acid	–	*A. lappa*	Roots	LCMS	Jaiswal and Kuhnert, 2011
159	1,5-dicaffeoyl-3-maloylquinic acid	–	*A. lappa*	Roots	LCMS	Jaiswal and Kuhnert, 2011
160	1,4-di-*O*-caffeoyl-3-*O*-maloylquinic Acid	C_29_H_27_O_16_	*A. lappa*	Roots	LCMS	Jaiswal and Kuhnert, 2011
161	1,3-di-*O*-caffeoyl-4,5-di-*O*-maloylquinic	C_33_ H_31_ O_20_	*A. lappa*	Roots	GCMS/LCMS	Jaiswal and Kuhnert, 2011; Tousch et al., 2014
162	1,5-dicaffeoyl-4-maloylquinic acid	–	*A. lappa*	Roots	LCMS	Jaiswal and Kuhnert, 2011
163	1,4-di-*O*-maloyl-3,5-di-*O*-caffeoylquinic acid	C_31_H_33_O_20_	*A. lappa*	Roots	GCMS/LCMS	Jaiswal and Kuhnert, 2011; Tousch et al., 2014
164	1,3,5-tricaffeoyl-4-succinoylquinic acid	–	*A. lappa*	Roots	LCMS	Jaiswal and Kuhnert, 2011
165	1,5-dicaffeoyl-3,4-disuccinoylquinic acid	–	*A. lappa*	Roots	LCMS	Jaiswal and Kuhnert, 2011
166	1,5-dicaffeoyl-3-fumaroyl-4-succinoylquinic acid	–	*A. lappa*	Roots	LCMS	Jaiswal and Kuhnert, 2011
167	1-fumaroyl-3,5-dicaffeoyl-4-succinoylquinic acid	–	*A. lappa*	Roots	LCMS	Jaiswal and Kuhnert, 2011
168	1,5-di-*O*-caffeoyl-3-*O*-succinoyl-4-*O*-maloylquinic acid	C_33_H_31_O_19_	*A. lappa*	Roots	GCMS/LCMS	Jaiswal and Kuhnert, 2011; Tousch et al., 2014
169	Dimaloyl-dicaffeoylquinic acid isomer 1	C_33_H_31_O_20_	*A. lappa*	Roots	GCMS/LCMS	Tousch et al., 2014
170	Succinoyl-tricaffeoylquinic acid isomer	C_38_H_33_O_18_	*A. lappa*	Roots	GCMS/LCMS	Tousch et al., 2014
171	Maloyl-dicaffeoylquinic acid isomer	C_29_H_27_O_15_	*A. lappa*	Roots	GCMS/LCMS	Tousch et al., 2014
172	Dicaffeoyl-succinoyl-malonylquinic acid isomer 1	C_33_H_31_O_19_	*A. lappa*	Roots	GCMS/LCMS	Tousch et al., 2014
173	Dicaffeoyl-succinoyl-malonylquinic acid isomer 2	C_33_H_31_O_20_	*A. lappa*	Roots	GCMS/LCMS	Tousch et al., 2014
174	Dimaloyl-dicaffeoylquinic acid isomer 2	C_33_H_31_O_20_	*A. lappa*	Roots	GCMS/LCMS	Tousch et al., 2014
175	Dimaloyl-dicaffeoylquinic acid isomer 3	C_33_H_31_O_20_	*A. lappa*	Roots	GCMS/LCMS	Tousch et al., 2014
176	Maloyl-tricaffeoylquinic isomer	C_28_H_32_O_19_	*A. lappa*	Roots	GCMS/LCMS	Tousch et al., 2014
177	1,3,5-tri-*O*-caffeoyl-4-*O*-maloylquinic Acid	C_38_H_33_O_19_	*A. lappa*	Roots	GCMS/LCMS	Tousch et al., 2014
178	5-hydroxymaltol	C_6_H_6_O_4_	*A. lappa*	Roots	UV/IR/ESIMS/NMR	Han et al., 2013
179	Succinic acid	C_4_H_6_O_4_	*A. lappa*	Roots	UV/IR/ESIMS/NMR	Han et al., 2013
	Saccharides/Polysaccharides					
180	Rhamnogalacturonan	C_117_H_178_O_101_	*A. lappa, A. minus*	Roots/leaves	Chromatography/NMR/sugar analysis	Kato and Watanabe, 1993; Carlotto et al., 2016
181	Xylan	(C_5_H_8_O_4_)n	*A. lappa, A. minus*	Roots/leaves	Chromatography/NMR/sugar analysis	Kato and Watanabe, 1993
182	Arabinan	C_9_H_13_N_3_O_5_	*A. lappa, A. minus*	Roots/leaves	Chromatography/ NMR/sugar analysis	Kato and Watanabe, 1993; Carlotto et al., 2016
183	Arabinogalactan	C_20_H_36_O_14_	*A. lappa, A. minus*	Roots/leaves	Chromatography/ NMR/sugar analysis	Kato and Watanabe, 1993; Carlotto et al., 2016
184	Galactan	C_18_H_32_O_16_	*A. lappa, A. minus*	Roots/leaves	Chromatography/ NMR/sugar analysis	Kato and Watanabe, 1993
185	Cellulose	C_64_H_124_O_30_	*A. lappa, A. minus*	Roots/leaves	Chromatography/ NMR/sugar analysis	Kato and Watanabe, 1993
186	Xyloglucan	C_51_H_86_O_42_	*A. lappa, A. minus*	Roots/leaves	Chromatography/ NMR/sugar analysis	Kato and Watanabe, 1993
187	Galacturonic acid	C_6_H_10_O_7_	*A. lappa*	Roots/leaves	Chromatography/ NMR	Carlotto et al., 2016
188	Galacturonic acid	C_6_H_10_O_7_	*A. lappa*	Roots	Chromatography	Fuchigami et al., 1990
189	Galactose	C_6_H_12_O_6_	*A. lappa*	Roots/leaves/fruits	Chromatography/ NMR	Fuchigami et al., 1990; Kardosova et al., 2003; Boldizsar et al., 2010; Carlotto et al., 2016
190	Glucose	C_6_H_12_O_6_	*A. lappa*	Roots/leaves/fruits	UV/NMR/HPLC/GCMS	Kardosova et al., 2003; Boldizsar et al., 2010; Li et al., 2013; Carlotto et al., 2016
191	Mannose	C_6_H_12_O_6_	*A. lappa*	Roots/leaves	NMR	Carlotto et al., 2016
192	Sucrose	C_12_H_22_O_11_	*A. lappa*	Roots	UV/NMR/HPLC/GCMS	Boldizsar et al., 2010; Li et al., 2013
193	Raffinose	C_18_H_32_O_16_	*A. lappa*	Fruits	UV/NMR/HPLC/GCMS	Boldizsar et al., 2010
194	Rhamnose	C_6_H_12_O_5_	*A. lappa*	Roots/leaves/fruits	UV/NMR/HPLC/GCMS	Boldizsar et al., 2010; Carlotto et al., 2016
195	Arabinose	C_5_H_10_O_5_	*A. lappa*	Roots/leaves/fruits	UV/NMR/HPLC/GCMS	Fuchigami et al., 1990; Kardosova et al., 2003; Boldizsar et al., 2010; Carlotto et al., 2016
196	Inulin (fructan)	(C6H10O5)n	*A. lappa, A. tomentosum*	Roots	HPTLC/MS/NMR/HPLC-ELSD	Kardosova et al., 2003; Turdumambetov et al., 2004; Milani et al., 2011; Olennikov and Tankhaeva, 2011; Li et al., 2013; Liu et al., 2014
197	Fructose	C_6_H_12_O_6_	*A. lappa*	Roots	HPLC-ELSD	Li et al., 2013
198	Sorbitol	C_6_H_14_O_6_	*A. lappa*	Fruits	UV/NMR/HPLC/GCMS	Boldizsar et al., 2010
199	Mannitol	C_6_H_14_O_6_	*A. lappa*	Fruits	UV/NMR/HPLC/GCMS	Boldizsar et al., 2010
	Others					
200	Crocin	C_44_H_64_O_24_	*A. lappa*	Leaves	UPLC	Lou et al., 2016
201	*b*-asparagine	C_4_H_8_N_2_O_3_	*A. lappa, A. tomentosum*	Roots	IR/NMR	Boev, 2005

#### Lignans

Major biologically active lignans include mainly arctigenin (a dietary phytoestrogen) and its glycoside, arctiin (lignanolides) occurring commonly in seeds, roots, fruits, and leaves of *A. lappa* and *A. tomentosum* (Yu et al., 2003; Ming et al., 2004; Liu et al., 2005, 2012, 2015; Wang et al., 2005; Matsumoto et al., 2006; Gao et al., 2008; Boldizsar et al., 2010; Ferracane et al., 2010; Zhou et al., 2011; Qin et al., 2014; Su et al., 2015; Lou et al., 2016). In addition, seeds and roots are distributed with low levels of dilignans and sesquilignans. For the first time, two new sesquilignans, namely lappaol A and B were isolated and characterized from *A. lappa* seeds (Ichihara et al., 1976). Later, 3 more sesquilignans, namely, lappaol C, D, and E, and two dilignans, namely lappaol F and H, were structurally determined from the seeds of *A. lappa* (Ichihara et al., 1977, 1978; Yong et al., 2007; Su et al., 2015). Lappaol A, C, and F are also found in the fruits of *A. tomentosum* (Kardosova et al., 2003). Two new lignans, neoarctin A and B, along with other recognized compounds including arctiin, arctigenin, daucosterol, lappaol F, isolappaol C and matairesinol were identified in seeds of *A. lappa* (Wang and Yang, 1995; Kardosova et al., 2003; Yong et al., 2007; Gao et al., 2008; Qin et al., 2014; Su et al., 2015). A simple RP-HPLC method was developed to identify the presence of arctiin in fruits of *A. lappa* (Yu et al., 2003; Boldizsar et al., 2010). Using bioactivity-guided fractionation, lappaol A, C and F, arctiin and arctignan E were isolated and characterized from the ethanolic extract (95%) of *A. lappa* seeds (Ming et al., 2004). Likewise, HPLC/UPLC/LC/MS/MS methods have been developed to identify arctigenin and arctiin in the seeds, leaves and roots of *A. lappa* (Yu et al., 2003; Liu et al., 2005; Ferracane et al., 2010; Lou et al., 2010a,b; Predes et al., 2011). Further, a supercritical fluid extraction procedure was found to be superior for extracting arctiin from *A. lappa* fruits (Carlotto et al., 2016). A high-speed counter-current chromatography was employed to obtain the pure compound arctiin from the fruit extracts of *A. lappa*. Authors obtained 49% of arctiin identified based on LC-MS and NMR techniques (Wang et al., 2005). A novel butyrolactone lignan compound named diarctigenin was found to occur in the methanolic seed extracts of *A. lappa* (Han et al., 1994). The fruits of *A. lappa* are reported to contain a total of 13 compounds including 5 new natural products (Umehara et al., 1993). Among them, 6 compounds were identified as arctignan A-E and artctigenin. Later, the occurrence of arctigenin and arctiin was also established from the leaves and seeds of *A. lappa* (Umehara et al., 1993; Liu et al., 2005; Matsumoto et al., 2006). Besides, the active extract resulted from the bioassay-guided fractionation of seed methanolic extract contained five active compounds including a new sesquilignan named isolappaol C and four known sesquilignan and dilignans namely, diarctigenin, and lappaol C, D, and F (Ferracane et al., 2010). Further, improved methods of extraction and analysis revealed that the seeds and roots of *A. lappa* contain arctigenin, arctiin, arctignan E, matareisinol, lappaol A, C, and F (Ferracane et al., 2010; Lou et al., 2010a, 2016; Liu et al., 2015; Su et al., 2015). The occurrence of 8 lignans in seeds and 1 lignan in roots of *A. lappa* was determined. The identified lignans were arctiin, arctigenin arctignan D and E, lappaol A, C, and H, isolappaol C and matairesinol (Liu et al., 2012; Haghi et al., 2013). Likewise, syringaresinol was reported in the chloroform fraction of *A. lappa* roots (Han et al., 2013). A rare butyrolactone lignan named arctiidilactone, and 11 novel butyrolactone lignans [arctigenin-4-*O*-β-D-gentiobioside, arctigenin-4-*O*-α-D-galactopyranosyl-(1→6)-*O*-β-D-glucopyranoside, arctigenin-4-*O*-β-D-apiofuranosyl-(1→6)-*O*-β-D-glucopyranoside, 5′-propanediolmatairesinoside, (7′R,8R,8′R)-rafanotrachelogenin-4-*O*-β-D-glucopyranoside, (7′S,8R,8′R)-rafanotrachelogenin-4-*O*-β-D-glucopyranoside, (7S,8S,8′R)-4,7-dihydroxy-3,3′, 4-trimethoxyl-9-oxo benzylbutyrolactone lignan-4-*O*-β-D-glucopyranoside, (7R,8S,8′R) -4,7,4′-trihydroxy-3,3′-dimethoxyl-9-oxo dibenzylbutyrolactone lignan-4-*O*-β-D-glucopyranoside, (7S,8S,8′R)-4,7-dihydroxy-3,3′,4′-trimethoxyl-9-oxo dibenzylbutyrolactone lignan, arctiidilactone, arctiiapolignan A and arctiisesquineolignan A] were determined in *A. lappa* fruits (Yang et al., 2015). Phylligenin, matairesinoside and pinoresinol were reported only in the fruits of *A. lappa* (Boldizsar et al., 2010). Also, 2 secolignans, styraxlignolide D and styraxlignolide E (Yang et al., 2015) and 4 new neolignan glucosides namely, (8R)-4,9,9′-trihydroxy-3,3′-dimethoxy-7-oxo-8-*O*-4′-neolignan-4-*O*-β-D-glucopyranoside, (7S,8R)-4,7,9,9′-tetrahydroxy-3,3′-dimethoxy-8-*O*-4′-neolignan-9′-*O*-β-D-apiofuranosyl-(1→6)-*O*-β-D-glucopyranoside, (7′S,8′R,8S)-4,4′, 9′-trihydroxy-3,3′-dimethoxy -7′,9-epoxylignan-7-oxo-4-*O*-β-D-glucopyranoside and (7R, 8S)-dihydrodehydrodiconiferyl alcohol-7′-oxo-4-*O*-β-D-glucopyranoside are reported from *A. lappa* fruits (Huang X.Y. et al., 2015). Besides, phytochemical analysis of *A. lappa* fruits revealed the existence of 2 more lignans named arctiisesquineolignan B and arctiiphenolglycoside A (He et al., 2016). Bioassay-guided separation and purification of hydroethanolic extracts of *A. lappa fruits* allowed to identify a new lignan, (+)-7,8-didehydroarctigenin along with arctigenin and matairesinol identified previously (Matsumoto et al., 2006).

#### Fatty Acids and Esters

In search of α-glucosidase inhibitory compounds, Miyazawa et al. (2005) found 11 compounds in *A. lappa* methanol extract. Among them, 10 compounds belonged to fatty acids. The identified compounds were linolenic acid, linoleic acid, methyl linoleate, methyl oleate, methyl linolenate, oleic acid, palmitic acid, methyl palmitate, methyl stearate, and stearic acid. Methanol extract from *Arctium lappa* L. which was found to contain sitosterol-β-D-glucopyranoside, methyl palmitate, methyl linoleate and methyl linoleneate showed an inhibitory activity against α-glucosidase at 97.3, 73.4, 66.5, and 68.5% respectively at a concentration of 200.0 μM (Miyazawa et al., 2005). Later, Kuo et al. (2012) identified methyl methyl α-linolenate, linolenic acid and methyl oleate as the chief constituents in the *n*-hexane fraction of *A. lappa* root (Kuo et al., 2012). The presence of linoleic acid, oleic acid, palmitic acid and stearic *acid* were also reported from *A. lappa* fruits (Boldizsar et al., 2010). Fatty acid composition of *A. tomentosum* seeds showed the occurrence of docosanoic acid, hexadecanoic acid, 9-hexadecenoic acid, 9,12-octadecadienoic acid, oxiraneoctanoic acid, eicosanoic acid, *cis*-13-eicosenoic acid, and tetracosanoic acid (Zhou et al., 2011).

#### Acetylenic Compounds

From the roots of *A. lappa*, Washino et al. (1986) isolated and characterized 9 sulfur-containing acetylenic compounds namely, arctinone-a, arctinone-b, arctinol-a, arctinol-b, arctinal, arctic acid-b, arctic acid-c, methyl arctate-b, and arctinone-a acetate. Based on the chemical and spectral analysis, it was found that all these compounds were derivatives of 5′- (1-propynyl)-2,2′-bithienyl-5-yl. Later, the occurrence of few guaianolides linked with a sulfur-containing acetylenic compounds namely dehydrodihydrocostus lactone, dehydrocostus lactone, lappaphen-a and lappaphen-b were discovered from the acetone extracts of *A. lappa* roots (Washino et al., 1986, 1987). Several bioactivities of the key *A. lappa* constituents have been well-described in literature including antibacterial and antifungal activities of acetylenic compounds (Takasugi et al., 1987) and anti-edematogenic activity on carrageenan-induced paw edema (Carlotto et al., 2016).

#### Phytosterols

Daucosterol, a natural phytosterol-like compound, was obtained from the seeds of *A*. *lappa* (Ahangarpour et al., 2017). The fruits of *A. tomentosum* are reported to contain 2 steroids, such as daucosterol and β-sitosterol. Using bioactivity-guided fractionation, daucosterol and β-sitosterol were recovered from the ethanolic extract (95%) of *A. lappa* seeds (Ming et al., 2004). Later, sitosterol-beta-D-glucopyranoside was found in the methanolic extracts of *A. lappa* (Miyazawa et al., 2005). Also, daucosterol and β-sitosterol compounds were detected from the chloroform extracts of *A. lappa* roots (Han et al., 2013). It was shown that phytosterol daucosterol inhibited cancer cell proliferation by inducing autophagy through reactive oxygen species-dependent manner (Zhao et al., 2015), and exhibited immunoregulatory activity by inducing protective Th1 immune response (Lee et al., 2007).

#### Polysaccharides

For the first time, Fuchigami et al. (1990; Ferracane et al., 2010) determined the pectic polysaccharides in edible *A. lappa* roots. Later investigations revealed the occurrence of several kinds of polysaccharides such as pectic substance, rhamnogalacturonan with neutral sugars, hemicellulose (arabinan, arabinogalactan, galactan, xylan, and xyloglucan), galacturonic acid, glucose, galactose, arabinose, rhamnose, mannose, and cellulose in cell walls of *A. lappa* and *A. minus* roots and leaves (Kato and Watanabe, 1993; Carlotto et al., 2016). Also, arabinose, glucose, galactose, rhamnose, and raffinose are reported from fruits of *A. lappa* (Boldizsar et al., 2010). The xyloglucan characterized from *A. minus* comprised a repeated unit of oligosaccharides of hepta-(Glc–Xyl = 4:3), deca-(Glc–Xyl–Gal–Fuc = 4:3:2:1) and nona-(Glc–Xyl–Gal–Fuc = 4:3:1:1) saccharides in the ratio of 14:5:12 (Kato and Watanabe, 1993). Biologically active inulin-type fructofuranans and other fructooligosaccharides have been identified from the roots of *A. lappa* (Kardosova et al., 2003). Inulin, a fiber comprising oligomers and polymers of fructose units linked by β(2→1) fructosyl–fructose bonds, has also been reported in the roots of *A. lappa* (Rajasekharan et al., 2015). A water-soluble polysaccharide fructan with a molecular weight of 4,600 Da, named as ALP1, was purified from *A. lappa* root and was composed of fructose and glucose in the molar ratio of 13:1. They were linked in →(1)-Fru*f*-(2)→, Fru*f*-(2)→ and Glc*p*-(1)→ (Liu et al., 2014). The structure was similar to the crude fructan obtained previously by Kardosova et al. (2003). In *A. tomentosum*, the glucofructans content is 24%, constituted by a polymer of 2 inulin type (GF-A and GF-B) and 1 graminan (a mixed type of glucofructans containing 1,2- and 2-6 bonds) type polysaccharides. HPTLC method was developed by Olennikov and Tankhaeva (2011) to quantify fructans in *A. tomentosum* and *A. lappa* (Olennikov and Tankhaeva, 2011). Two sugar alcohols, mannitol and sorbitol were reported from the fruits of *A. lappa* (Boldizsar et al., 2010). The yield of inulin from *A. lappa* root was successfully increased by adopting an ultrasonic extraction technology (Milani et al., 2011). It was indicated that water-soluble polysaccharide from *A. lappa* could significantly ameliorate the dysregulation of pro-inflammatory cytokines (IL-1β, IL-6, and TNF-α) and anti-inflammatory cytokine (IL-10) caused by colitis (Wang et al., 2019).

#### Caffeoylquinic Acid Derivatives (Carboxylic Acids)

Caffeoylquinic acids are the major bioactive phenolic compounds of *Arctium* species and impart superior antioxidant properties to the plant. The roots of *A. lappa* were reported to contain caffeoylquinic acid derivatives such as 1-0-,5-*O*-dicaffeoylquinic acid, 1-0-,5-*O*-dicaffeoyl-3-*O*-succinylquinic acid, 1-0,-5-*O*-dicaffeoyl-4-*O*-succinylquinic acid, 1-0-,5-*O*-dicaffeoyl-3-*O*-,4-*O*-disuccinylquic acid and 1-0-,3-0-,5-*O*-tricaffeoyl-4-*O*- succinylquinic acid (Maruta et al., 1995). Chlorogenic acid content is much higher than the caffeic acid and both occur mainly in the skin of *A. lappa* roots (Chen et al., 2004). HPTLC analysis was used as a chemical profiling tool to estimate chlorogenic acid in *A. lappa* roots. The content ranged from 0.107 to 0.140%. Lin and Harnly (2008) and Liu et al. (2012) identified several compounds, including 5-sinapoylquinic acid, 3-sinapoyl-5-caffeoylquinic acid, 3-sinapoyl-5-caffeoyl-1- methoxyoxaloylquinic acid, 4-sinapoyl-5-caffeoyl-1-methoxyoxaloylquinic acid, 1,4-dicaffeoylquinic acid, 3,4-dicaffeoylquinic acid, 4,5-dicaffeoylquinic acid, 3,5-dicaffeoylquinic acid, 3,5-dicaffeoyl-1-methoxyoxaloylquinic acid, 3-feruloyl- 5-caffeoylquinic acid, 4,5-dicaffeoyl-1-methoxyoxaloylquinic acid, 3,5-dicaffeoyl-1- methoxyoxaloylquinic acid, 3-feruloyl-5-caffeoylquinic acid, 4,5-dicaffeoyl-1- methoxyoxaloylquinic acid, 3,4,5-tricaffeoylquinic acid, 1,4,5-tricaffeoylquinic acid and 1,4,5-tricaffeoyl-3-methoxyoxaloylquinic acid from the roots of *A. lappa*. Jaric et al. (2007) and Jaiswal and Kuhnert (2011) have characterized succinic, fumaric and malic acid-containing chlorogenic acid from the roots of *A. lappa*. These compounds included 3-succinoyl-4,5-dicaffeoyl, 1,5-dicaffeoyl-4-succinoylquinic acid, 1,5-dicaffeoyl-3-succinoylquinic acid, 3,4-dicaffeoyl-5-succinoylquinic acid, 1,5-dicaffeoyl-4-fumaroylquinic acid, 1,3-dicaffeoyl-5-fumaroylquinic acid, 1,4-dicaffeoyl-3-maloylquinic acid, 1,5-dicaffeoyl-3-maloylquinic acid and 1,5-dicaffeoyl-4-maloylquinic acid, 1,3,5-tricaffeoyl-4-succinoylquinic acid, 1,5-dicaffeoyl-3,4-disuccinoylquinic acid, 1,5-dicaffeoyl-3-fumaroyl-4-succinoylquinic acid, 1-fumaroyl-3,5-dicaffeoyl-4-succinoylquinic acid, 1,5-dicaffeoyl-3-succinoyl- 4-dimaloylquinic acid and dicaffeoyldimaloylquinic acid. Further, Liu et al. (2012) isolated and identified 12 caffeoylquinic acids in both seeds and roots of *A. lappa*. The identified compounds included chlorogenic acid, 1,5-di-*O*-caffeoylquinic acid, 1,3-di-*O*-caffeoylquinic acid, dicaffeoyl-maloylquinic acid, dicaffeoyl-maloylquinic acid, 1,3-di-*O*-caffeoylquinic acid, 1,5-di-*O*-caffeoyl-3-*O*-maloylquinic acid, 1,5-di-*O*-caffeoyl-3-*O*-succinoylquinic acid, 1,5-di-*O*-caffeoyl-4-*O*-maloylquinic acid, dicaffeoyl-dimaloylquinic acid, 1,5-di-*O*-caffeoylquinic acid, 1,5-di-*O*-caffeoyl-3-*O*-succinoyl-4-Omaloyquinic acid, 1,5-di-*O*-caffeoyl-3,4-di-*O*-succinoylquinic acid, and 1,3,5-tri-*O*-caffeoyl-4-*O*-succinoylquinic acid. In addition, phytochemical analysis of root extracts of *A. lappa* showed the occurrence of 8 additional isomers of hydroxycinnamic acids (Liu et al., 2012; Tousch et al., 2014). An average content of chlorogenic acid, 1-*O*-5-*O*-dicaffeoylquinic acid and 1,5-dicaffeoyl- 3-succinylquinic acid was observed to be between 1.7 and 7.9 mg/g dry weight of roots (Wang et al., 2001). Two new neolignan glucosides named (70S, 80R, 8S)-4,40,90- trihydroxy-3,30-dimethoxy-70,9-epoxylignan-7-oxo-4-*O*-b-D-glucopyranosyl-40-*O*-b-D-glucopyranoside and (7S, 8R)-4,7,9,90-tetrahydroxy-3,30-dimethoxyl- 70-oxo-8-40- oxyneolignan-4-*O*-b-D-glucopyranoside were determined from the fruit extract of *A. lappa* (Yang et al., 2012). The occurrence of phenolic acids, caffeic acid, cynarin and chlorogenic acid has been reported for the first time in *A. lappa* seeds and leaves (Ferracane et al., 2010; Tang et al., 2014; Lou et al., 2016) and later in both seeds and roots (Predes et al., 2011). Chlorogenic acid was determined by Tardio et al. (2005) in the seeds of *A. lappa*. Further, UPLC analysis revealed the presence of caffeic acid, benzoic acid and *p*-coumaric acid in the leaves of *A. lappa* (Tardio et al., 2005; Lou et al., 2010a,b). From the ethyl acetate and *n*-butanol fractions of *A. lappa*, 1,5-*O*-two caffeoylquinic acids, succinic acid and 5-hydroxy maltol were identified for the first time (Han et al., 2013). Likewise, HPLC and UPLC with photodiode array (PDA) detector were used to quantify caffeoyl esters, chlorogenic acid and 1,5-dicaffeoylquinic acid in aerial parts and root samples of *A. lappa* (Haghi et al., 2013). Two more phenolic compounds, namely, coumaroylquinic acid and caffeoyl-hexose-hydroxyphenol were identified by Rajasekharan et al. (2015) in the root extracts of *A. lappa* (Rajasekharan et al., 2015). It was reported that caffeoylquinic acids and their derivatives show multiple pharmacological activities including decrease in diet-induced obesity *via* modulation of PPARα and LXRα transcription (Huang K. et al., 2015) and anti-ulcerogenic effect (Lee et al., 2010).

#### Flavonoids

The reported flavonoids include flavonols, flavones, and their glycosides. Two major constituents, namely rutin and isoquercetin, along with few other minor flavonoids including kaempferol-3-*O*-rhamnoglucoside, quercimeritrin and astragalin were identified in the ethanolic extracts of *A. minus* leaves (Saleh and Bohm, 1971). Likewise, the occurrence of quercetin-3-*O*-rhamnoside was reported from the leaves of *A. lappa*. Later, the presence of phenolic compounds such as quercetin, quercitrin, rutin, and luteolin have been reported in seeds, fruits, leaves and roots of *A. lappa* (Saleh and Bohm, 1971; Tamayo et al., 2000; Yu et al., 2003; Lou et al., 2010a,b, 2016; Predes et al., 2011; Liu et al., 2012; Tang et al., 2014). Also, few isoflavone derivatives including genistein, nobilein, biachanin A and tangeretin have been detected in *A. lappa* roots (Eberding et al., 2007). A comparative study has shown the existence of chemical differences within the *A. lappa* organs (Ferracane et al., 2010). According to them, luteolin and quercetin rhamnoside were detected in roots whereas rutin, quercetin, quercitrin and luteolin in leaves. On the other hand, no flavonoids were found in the seeds of *A. lappa*. Two more flavonols, namely quercetin 3-*O*-glucuronide and quercetin 3-vicianoside, were identified by Rajasekharan et al. (2015) in the root extracts of *A. lappa*.

#### Terpenoids

The fruits of *A. lappa* were found to contain β-eudesmol, a sesquiterpene alcohol (Rajasekharan et al., 2015; Yang et al., 2015). Pentacyclic triterpenoids, such as ursolic and oleanolic acids were detected by Han et al. in the ethanolic extract of *A. lappa* roots (Han et al., 2013). Arctiopicrin and onopordopicrin are the sesquiterpene lactones isolated from the leaf extract of *A. lappa* (Barbosa et al., 1993; Machado et al., 2012). Arctiopicrin occurrence is also evidenced in *A. lappa*. Later, few more sesquiterpene lactones, namely dehydromelitensin-8- (4′-hydroxymethacrylate), dehydromelitensin, and melitensin and a norisoprenoid along with 2 more terpenes such as dehydrovomifoliol and loliolide were identified in *A. lappa* leaf (Machado et al., 2012). Onopordopicrin, a germacranolide sesquiterpene lactone was isolated from the aerial parts of *A. nemorosum* (Zimmermann et al., 2012). Two triterpenoids, namely 3α-acetoxy-hop-22(29)-ene and 3α-hydroxylanosta-5, 15-diene were isolated from the leaves of *A. lappa* (Jaiswal and Kuhnert, 2011).

#### Others

From the concentrated sap obtained from *A. lappa* roots (*A. lappa* and *A. tomentosum*), β-asparagine was isolated for the first time (Boldizsar et al., 2010). The carotenoid crocin was reported to occur in the leaves of *A. lappa* (Lou et al., 2016).

### Volatile Compounds

A total of 101 volatile chemical constituents were identified in *A. lappa.* The details of these compounds are partially summarized in Table 2 and described in this section. Carboxylic acids and fatty acids were more prevalent in *A. lappa.* On the other hand, there are no available literatures on the identification of volatile components in other *Arctium* species. The chemical structures of some compounds from *Arctium* species are shown in Figure 2.

**Table 2:** Volatile constituents of *Arctium* spp.

S. No	Compound name	Formula	Species	Plant origin/part	Analytical method	References
	Hydrocarbons					
1	Aplotaxene	C_17_H_28_	*A. lappa*	Roots	GCMS	Washino et al., 1986, 1987
2	Clovene	C_15_H_24_	*A. lappa*	Roots	GCMS	Washino et al., 1986, 1987
3	Dihydroaplotaxene	C_17_H_30_	*A. lappa*	Roots	GCMS	Washino et al., 1986, 1987
4	Docosane	C_22_H_46_	*A. lappa*	Leaves	GCMS	Aboutabl et al., 2013
5	Eicosane	C_20_H_42_	*A. lappa*	Roots/leaves/seeds	GCMS	Aboutabl et al., 2013
6	1-Heptadecene	C_17_H_34_	*A. lappa*	Roots	GCMS	Washino et al., 1986, 1987
7	Heptacosane	C_27_H_56_	*A. lappa*	Roots/leaves	GCMS	Aboutabl et al., 2013
8	Hexacosane	C_26_H_54_	*A. lappa*	Roots/leaves	GCMS	Aboutabl et al., 2013
9	Nonadecane	C_19_H_40_	*A. lappa*	Leaves	GCMS	Aboutabl et al., 2013
10	2-Naphthalenemethanol	C_11_H_10_O	*A. lappa*	Roots	GCMS	Wang et al., 2004
11	1-Pentadecene	C_15_H_30_	*A. lappa*	Roots	GCMS	Washino et al., 1986, 1987
12	Pentacosane	C_25_H_52_	*A. lappa*	Roots	GCMS	Aboutabl et al., 2013
13	Pentadecane	C_15_H_32_	*A. lappa*	Roots/leaves	GCMS	Aboutabl et al., 2013
14	Tetracosane	C_24_H_50_	*A. lappa*	Roots/leaves	GCMS	Aboutabl et al., 2013
15	Tetradecane	C_14_H_30_	*A. lappa*	Leaves	GCMS	Aboutabl et al., 2013
	Aldehydes					
16	Benzaldehyde	C_7_H_6_O	*A. lappa*	Roots	GCMS	Washino et al., 1986, 1987; Wang et al., 2004
17	Butanal	C_4_H_8_O	*A. lappa*	Roots	GCMS	Washino et al., 1986, 1987
18	Decanal	C_10_H_20_O	*A. lappa*	Roots	GCMS	Washino et al., 1986, 1987
19	Dodecanal	C_12_H_24_O	*A. lappa*	Roots	GCMS	Washino et al., 1986, 1987
20	Heptanal	C_7_H_14_O	*A. lappa*	Roots	GCMS	Washino et al., 1986, 1987
21	Hexanal	C_6_H_12_O	*A. lappa*	Roots	GCMS	Washino et al., 1986, 1987
22	(*Z*)-3-Hexenal	C_6_H_10_O	*A. lappa*	Roots	GCMS	Washino et al., 1986, 1987
23	(*E*)-2-Hexenal	C_6_H_10_O	*A. lappa*	Roots	GCMS	Washino et al., 1986, 1987
24	2-Methylpropanal	C_4_H_8_O	*A. lappa*	Roots	GCMS	Washino et al., 1986, 1987
25	3-Methylbutanal	C_5_H_10_O	*A. lappa*	Roots	GCMS	Washino et al., 1986, 1987
26	Nonanal	C_9_H_18_O	*A. lappa*	Roots/leaves/seeds	GCMS	Washino et al., 1986, 1987
27	Octanal	C_8_H_16_O	*A. lappa*	Roots	GCMS	Washino et al., 1986, 1987
28	(*E*)-2-Octanal	C_8_H_14_O	*A. lappa*	Roots	GCMS	Washino et al., 1986, 1987
29	Phenylacetaldehyde	C_8_H_8_O	*A. lappa*	Roots	GCMS	Washino et al., 1986, 1987
30	Pentanal	C_5_H_10_O	*A. lappa*	Roots	GCMS	Washino et al., 1986, 1987
31	Propanal	C_3_H_6_O	*A. lappa*	Roots	GCMS	Washino et al., 1986, 1987
32	Tridecanal	C_13_H_26_O	*A. lappa*	Roots	GCMS	Washino et al., 1986, 1987
33	4-Methoxybenzaldehyde	C_8_H_8_O_2_	*A. lappa*	Roots	GCMS	Washino et al., 1986, 1987
34	Undecanal	C_11_H_22_O	*A. lappa*	Roots		Washino et al., 1986, 1987
	Methoxypyrazines					
35	2-Methoxy-3-methylpyrazine	C_6_H_8_N_2_O	*A. lappa*	Roots	GCMS	Washino et al., 1986, 1987
36	2-Isopropyl- 3-methyoxylpyrazine	C_8_H_12_N_2_O	*A. lappa*	Roots	GCMS	Washino et al., 1986, 1987
37	2-Methoxy-3- propylpyrazine	C_8_H_12_N_2_O	*A. lappa*	Roots	GCMS	Washino et al., 1986, 1987
38	2-sec-Butyl-3-methoxypyrazine	C_9_H_14_N_2_	*A. lappa*	Roots	GCMS	Washino et al., 1986, 1987
39	2-Isobutyl-3-methoxypyrazine	C_9_H_14_N_2_O	*A. lappa*	Roots	GCMS	Washino et al., 1986, 1987
40	2-Butyl-3- methoxypyrazine	C_9_H_14_N_2_	*A. lappa*	Roots	GCMS	Washino et al., 1986, 1987
41	2-Isoamyl-3-methoxypyrazine	C_9_H_14_N_2_O	*A. lappa*	Roots	GCMS	Washino et al., 1986, 1987
	Fatty acids/Carboxylic acids					
42	Acetic acid	CH_3_COOH	*A. lappa*	Roots	GCMS	Washino et al., 1986, 1987
43	Benzoic acid	C_7_H_6_O_2_	*A. lappa*	Roots	GCMS	Washino et al., 1986, 1987
44	Butyric acid	C_4_H_8_O_2_	*A. lappa*	Roots	GCMS	Washino et al., 1986, 1987
45	Cinnamic acid	C_9_H_8_O_2_	*A. lappa*	Roots	GCMS	Washino et al., 1986, 1987
46	Costic acid	C_15_H_22_O_2_	*A. lappa*	Roots	GCMS	Washino et al., 1986, 1987
47	Decanoic acid	C_10_H_20_O_2_	*A. lappa*	Roots	GCMS	Washino et al., 1986, 1987
48	Dodecanoic acid	C_12_H_24_O_2_	*A. lappa*	Roots	GCMS	Washino et al., 1986, 1987
49	Ethyl oleate	C_20_H_38_O_2_	*A. lappa*	Seeds	GCMS	Aboutabl et al., 2013
50	Hexanoic acid	C_3_H_6_O_2_	*A. lappa*	Roots	GCMS	Washino et al., 1986, 1987
51	Hexadecanoic acid	C_17_H_34_O_2_	*A. lappa*	Roots/seeds	GCMS	Washino et al., 1986, 1987; Aboutabl et al., 2013
52	(*E*)-3-Hexenoic acid	C_6_H_10_O_2_	*A. lappa*	Roots	GCMS	Washino et al., 1986, 1987
53	Heptanoic acid	C_7_H_14_O_2_	*A. lappa*	Roots	GCMS	Washino et al., 1986, 1987
54	(*E*)-3-Heptenoic acid	C_7_H_12_O_2_	*A. lappa*	Roots	GCMS	Washino et al., 1986;, 1987
55	Linoleic acid	C_18_H_32_O_2_	*A. lappa*	Roots	GCMS	Wang et al., 2004
56	2, 3-Hydroxyoctanoic acid	C_8_H_16_O_3_	*A. lappa*	Roots	GCMS	Washino et al., 1986, 1987
57	2-Methylpropionic acid	C_4_H_8_O_2_	*A. lappa*	Roots	GCMS	Washino et al., 1986, 1987
58	2-Methylbutyric acid	C_5_H_10_O_2_	*A. lappa*	Roots	GCMS	Washino et al., 1986, 1987
59	3-Methoxybenzoic acid	C_8_H_8_O_3_	*A. lappa*	Roots	GCMS	Washino et al., 1986, 1987
60	Methyl palmitate	C_17_H_34_O_2_	*A. lappa*	Roots/seeds	GCMS	Washino et al., 1986, 1987; Aboutabl et al., 2013
61	Methyl linolenate	C_19_H_32_O_2_	*A. lappa*	Roots	GCMS	Wang et al., 2004
62	Methyl oleate	C_19_H_36_O_2_	*A. lappa*	Seeds	GCMS	Aboutabl et al., 2013
63	Nonanoic acid	C_9_H_18_O_2_	*A. lappa*	Roots	GCMS	Washino et al., 1986, 1987
64	Nonanedioic acid	C_9_H_16_O_4_	*A. lappa*	Roots	GCMS	Washino et al., 1986;, 1987
65	(*E*)-3-nonenoic acid	C_9_H_16_O_2_	*A. lappa*	Roots	GCMS	Washino et al., 1986, 1987
66	Octanoic acid	C_8_H_16_O_2_	*A. lappa*	Roots	GCMS	Washino et al., 1986, 1987
67	(*E*)-3-Octenoic acid	C_8_H_14_O_2_	*A. lappa*	Roots	GCMS	Washino et al., 1986, 1987
68	Octadecanoic acid	C_18_H_36_O_2_	*A. lappa*	Roots	GCMS	Washino et al., 1986, 1987
69	Octadecanoic acid methyl ester	C_18_H_36_O_2_	*A. lappa*	Seeds	GCMS	Aboutabl et al., 2013
70	Pentanoic acid	C_5_H_10_O_2_	*A. lappa*	Roots	GCMS	Washino et al., 1986, 1987
71	Phenylacetic acid	C_8_H_8_O_2_	*A. lappa*	Roots	GCMS	Washino et al., 1986, 1987
72	Phenylpropionic acid	C_9_H_10_O_2_	*A. lappa*	Roots	GCMS	Washino et al., 1986, 1987
73	Propionic acid	C_3_H_6_O_2_	*A. lappa*	Roots	GCMS	Washino et al., 1986, 1987
74	Pentadecanoic acid	C_15_H_30_O_2_	*A. lappa*	Roots	GCMS	Washino et al., 1986, 1987
75	Salicylic acid	C_7_H_6_O_3_	*A. lappa*	Roots	GCMS	Washino et al., 1986, 1987
76	Tridecanoic acid	C_13_H_26_O_2_	*A. lappa*	Roots	GCMS	Washino et al., 1986, 1987
77	Tetradecanoic acid	C_14_H_28_O_2_	*A. lappa*	Roots	GCMS	Washino et al., 1986, 1987
78	Undecanoic acid	C_11_H_22_O_2_	*A. lappa*	Roots	GCMS	Washino et al., 1986, 1987
	Terpenes/terpenoids					
	Monoterpenoids					
79	Carvomenthone	C_10_H_18_O	*A. lappa*	Roots/leaves	GCMS	Aboutabl et al., 2013
80	Geraniol	C_10_H_18_O	*A. lappa*	Seeds	GCMS	Aboutabl et al., 2013
81	Linalool	C_10_H_18_O	*A. lappa*	Seeds	GCMS	Aboutabl et al., 2013
82	Thymol	C_10_H_14_O	*A. lappa*	Seeds	GCMS	Aboutabl et al., 2013
83	*Z*-citral	C_10_H_16_O	*A. lappa*	Seeds	GCMS	Aboutabl et al., 2013
84	*E*-citral	C_10_H_16_O	*A. lappa*	Seeds	GCMS	Aboutabl et al., 2013
	Sesquiterpenoids					
85	Dehydrocostus lactone	C_15_H_18_O_2_	*A. lappa*	Roots	GCMS	Washino et al., 1986, 1987
86	Dehydrodihydrocostus lactone	C_15_H_29_O_2_	*A. lappa*	Roots	GCMS	Washino et al., 1986, 1987
	Oxygenated sesquiterpenes					
87	Caryophyllene oxide	C_15_H_24_O	*A. lappa*	Roots/leaves	GCMS	Aboutabl et al., 2013
88	β-Costol	C_15_H_24_O	*A. lappa*	Roots	GCMS	Aboutabl et al., 2013
	Sesquiterpene Hydrocarbons					
89	Aromadendrene	C_15_H_24_	*-*	Roots/seeds	GCMS	Aboutabl et al., 2013
90	Caryophyllene	C_15_H_24_	*A. lappa*	Roots	GCMS	Washino et al., 1986, 1987
91	γ-Cadinene	C_15_H_24_	*-*	Roots/leaves/seeds	GCMS	Aboutabl et al., 2013
92	Cyperene	C_15_H_24_	*A. lappa*	Roots	GCMS	Washino et al., 1986, 1987
93	β-Elemene	C_15_H_24_	*A. lappa*	Roots	GCMS	Washino et al., 1986, 1987; Aboutabl et al., 2013
94	*trans*-β-Farnesene	C_15_H_24_	*A. lappa*	Roots/leaves	GCMS	Aboutabl et al., 2013
95	α-Guaiene	C_15_H_24_	*A. lappa*	Roots	GCMS	Washino et al., 1986, 1987
96	Isoaromadendrene epoxide	C_15_H_24_O	*–*	Roots/leaves/seeds	GCMS	Aboutabl et al., 2013
97	Limonene	C_10_H_16_	*A. lappa*	Leaves/seeds	GCMS	Washino et al., 1986, 1987
98	α-Myrcene	C_10_H_16_	*A. lappa*	Seeds	GCMS	Aboutabl et al., 2013
99	α-Pinene	C_10_H_16_	*A. lappa*	Roots/leaves	GCMS	Aboutabl et al., 2013
100	Squalene	C_30_H_50_	*A. lappa*	Seeds	GCMS	Aboutabl et al., 2013
	Sesquiterpene Alcohol					
101	β-Copaen-4α-ol	C_15_H_24_O	–	Roots/leaves/seeds	GCMS	Aboutabl et al., 2013

#### Hydrocarbons

Fourteen hydrocarbon compounds, aplotaxene, clovene, dihydroaplotaxene, eicosane, 1-heptadecene, heptacosane, hexacosane, nonadecane, 2-naphthalenemethanol, 1-pentadecene, pentacosane, pentadecane, tetracosane, and tetradecane were detected from the roots, seeds, and leaves of *A. lappa* (Washino et al., 1986; Wang et al., 2004). In addition, docosane, eicosane, heptacosane, hexacosane, tetracosane, and pentadecane were found only in roots and leaves. Docosane was found only in leaves, while seeds of *A. lappa* contained only eicosane.

#### Aldehydes

Nineteen aldehydes, namely, benzaldehyde, butanal, decanal, dodecanal, heptanal, hexanal, (*Z*)-3-hexenal, (*E*)-2-hexenal, 2-methylpropanal, 3-methylbutanal, nonanal, octanal, (*E*)-2-octanal, phenylacetaldehyde, pentanal, propanal, tridecanal, 4-methoxybenzaldehyde, and undecanal were found as root volatile compounds in *A. lappa* (Washino et al., 1986, 1987; Wang et al., 2004). Interestingly, only the alkyl aldehyde nonanal was present in all plant parts such as roots, leaves, and seeds (Washino et al., 1986, 1987).

#### Methoxypyrazines

Seven methoxypyrazines, such as 2-methoxy-3-methylpyrazine, 2-methoxy-3- propylpyrazine, 2-isopropyl- 3-methyoxylpyrazine, 2-sec-butyl-3-methoxypyrazine, 2-butyl-3- methoxypyrazine, 2-isobutyl-3-methoxypyrazine, and 2-isoamyl-3-methoxypyrazine were detected in roots of *A. lappa* (Washino et al., 1986, 1987).

#### Carboxylic Acids and Fatty Acids

Twenty-two carboxylic acids namely acetic acid, benzoic acid, butyric acid, cinnamic acid, costic acid, dodecanoic acid, hexanoic acid, (*E*)-3-hexenoic acid, heptanoic acid, (*E*)-3-heptenoic acid, 2, 3-hydroxyoctanoic acid, 2-methylpropionic acid, 2-methylbutyric acid, 3-methoxybenzoic acid, nonanoic acid, nonanedioic acid, pentanoic acid, phenylacetic acid, phenylpropionic acid, propionic acid, salicylic acid, and undecanoic acid were identified in *A. lappa* roots (Washino et al., 1986, 1987; Wang et al., 2004). Fatty acids such as decanoic acid, hexadecanoic acid, linoleic acid, octanoic acid, (*E*)-3-octenoic acid, octadecanoic acid, pentadecanoic acid, tridecanoic acid, and tetradecanoic acid were found in roots while ethyl oleate, methyl oleate, hexadecanoic acid, methyl palmitate, and octadecanoic acid methyl ester were identified in seeds of *A. lappa* (Washino et al., 1986, 1987; Wang et al., 2004).

#### Monoterpenes and Sesquiterpenes

Three alcoholic and one phenolic monoterpenoids (carvomenthone, geraniol, linalool, and thymol); 2 sesquiterpene lactones (dehydrocostus lactone and dehydrodihydrocostus lactone, isoaromadendrene epoxide); 2 oxygenated sesquiterpenes (caryophyllene oxide and β-costol) and 12 sesquiterpene hydrocarbons namely, aromadendrene, caryophyllene, γ-cadinene, cyperene, β-elemene, *trans*-β-farnesene, α-guaiene, limonene, myrcene, α-pinene, and squalene were identified in *A. lappa* (Washino et al., 1986, 1987; Wang et al., 2004). Geraniol, linalool, thymol, aromadendrene, γ-cadinene, isoaromadendrene epoxide, limonene, α-myrcene, and squalene were identified only in the seeds of *A. lappa.*α-Pinene, isoaromadendrene epoxide, γ-cadinene, carvomenthone, and caryophyllene oxide were found in the roots and leaves.

## Bioactivities of *Arctium* Species

*Arctium lappa* is widely used as an ethno-medicinal plant especially in North America, Asia and Europe, and is applied to treat various diseases including diabetes, gout, rheumatism, and skin problems (Chan et al., 2011; Azizov et al., 2012). *A. lappa* roots have been used as a vegetable in Japanese (referred to as ‘gobo’) and Korean cuisine. Its root has been used to treat constipation, mercury poisoning, upper respiratory infections, inflammation and oxidative stress in patients with knee osteoarthritis (Maghsoumi-Norouzabad et al., 2016), while the leaves were efficacious in healing burns, rashes, and applied in women with labor condition (Force, 2001; Lewis and Elvin-Lewis, 2003; Amish Burn Study et al., 2014). *A. lappa* has also been found for the treatment of alopecia (loss of hair) among adults (Amish Burn Study et al., 2014). In Western countries, burdock is used as a remedy for several ailments ranging from arthritis, chronic inflammation, and various skin problems (e.g., scaly skin conditions such as psoriasis and eczema) to cancer treatment (Wu et al., 2010; Amish Burn Study et al., 2014).

Studies on the biological activities of extracts of different parts of *A. lappa* and compounds isolated thereof, were carried out and revealed antipyretic, antimicrobial, diuretic, diaphoretic, hypoglycaemic, antioxidant, anti-inflammatory, anti-hepatotoxicity, antiulcer, antimutagenicity, and antitumour activities.

### Anticancer Effects

*Arctium lappa* fruit has been used in traditional medicine, and it is popular for its various anticancer effects. Arctigenin (ATG), a natural lignan product extracted from the seeds of *Arctium lappa*, has been shown to have estrogenic properties, that reduced the risk of osteoporosis, heart disease, and menopausal symptoms (Maxwell et al., 2017). It was found to possess antitumor effect by modulating the protein kinase activation pathway and hence rendering the tumor cells susceptible to effects of the nutrient-deprived environment (Awale et al., 2006). Later on, ATG was shown to induce apoptosis (programmed cell death) of estrogen receptor-negative cancer cells (MDA-MB-231) through the ROS/p38 MAPK pathway and epigenetic regulation of Bcl-2 by upregulating trimethylation of histone H3K9 (Hsieh et al., 2014). It was reported that ATG was able to inhibit cell proliferation and may induce apoptosis and cell cycle arrest at the G0/G1 phase in glioma cells (Maimaitili et al., 2017). In more detail, it was found that ATG increased the expression levels of p21, retinoblastoma and p53 proteins, and significantly decreased the expression levels of cyclin D1 and CDK4 proteins (Maimaitili et al., 2017). Furthermore, ATG was able to induce apoptosis in glioma cells, coupled with increased expression levels of cleaved caspase-3 and the pro-apoptotic BCL2-associated X protein (Maimaitili et al., 2017). ATG-induced apoptosis was significantly suppressed by the pretreatment of cells with Z-DEVD-FMK, a caspase-3 inhibitor (Maimaitili et al., 2017). More recently, study by Lou et al. (2017) demonstrated ATG to significantly inhibit *in vitro* migration and invasion of human breast cancer cells (MDA-MB-231) by downregulation of MMP-2, MMP-9 and heparanase (Lou et al., 2017).

Extracts from *A. lappa* also showed selective antiproliferative activity against certain human cancer cell lines including K562, MCF-7 and 786-0 (Predes et al., 2011). Lappaol F, a novel natural product isolated from the seeds of *A. lappa*, was found to suppress cancer cell growth in a dose-dependent manner in various human cancer cell lines through induction of G1 and G2 cell-cycle arrest. This effect was associated with strong induction of p21 and p27 and suppression of cyclin-dependent kinase 1 (CDK1) and cyclin B1 (Sun et al., 2014).

*A. lappa* is one of the herbs widely used by cancer patients in some Canadian populations to improve quality of life (QOL) and prevent cancer progression. *A. lappa* is one of the herbs constituting the two proprietary herbal products: Flor-Essence^®^ and Essiac^®^ suggested for prolong survival and the improvement of QOL among cancer patients (Tamayo et al., 2000).

### Antidiabetic Effects

Root of *A. lappa* root has been found to mediate hypoglycemic activities making it a popular choice to be used as a traditional medicine in diabetes. Oral administration of burdock root ethanolic extract in streptozotocin-induced diabetic rats significantly lowered blood glucose and increased insulin level in the diabetic rats compared to the control diabetic group (Cao et al., 2012). Additionally, treatment with *A. lappa* extract also reduced the levels of serum total cholesterol (TC), triglycerides (TG) and low density lipoprotein (LDL), whereas high density lipoprotein (HDL) level was higher in the control rats. More recently in a similar study, Ahangarpour et al. (2017) investigated the antidiabetic and hypolipidemic properties of the root extract of *A. lappa* on nicotinamide-streptozotocin (NA-STZ)-induced type 2 diabetes in mice (Ahangarpour et al., 2017). The results show that root extract of *A. lappa* displays anti-diabetic effect at certain doses. It exerts its effects through hypolipidemic and insulinotropic properties and hence the root extract could serve successfully in treating patients with type 2 diabetes in the future. Moreover, sitosterol-β-D-glucopyranoside from burdock’s root acts as a potent inhibitor of alpha-glucosidases, thereby having the potential to reduce glycogenolysis and help to decrease blood glucose level (Tousch et al., 2014). In addition, Zhao and Zhou (2015) demonstrated that trace elements (e.g., Na, K, Mn, Fe, and Mg) present in the root and fruit extracts of *A. lappa* exhibit antidiabetic effects. While *A. lappa* constituents do reduce absorption of glucose, they also elevate inulin content in blood and slow digestion of carbohydrates to confer its anti-diabetic activities. The pharmacological mechanisms of *A. lappa* roots are slightly different from other classes of oral antihyperglycemic agents such as metformin. Metformin decreases hepatic glucose production, decreases intestinal absorption of glucose, and improves insulin sensitivity by increasing peripheral glucose uptake and utilization (Dumitrescu et al., 2015).

### Anti-oxidant, Hepatoprotective and Gastroprotective Activities

It is believed that lignans and caffeoylquinic acids from *A. lappa* are of value because of their antioxidant capacity (Maruta et al., 1995; Mkrtchian et al., 1998; Jaiswal and Kuhnert, 2011) by which they can scavenge free radicals that are thought to play an important role in many diseases.

The hydroalcoholic extracts of burdock roots possess significant antioxidant potential as seen by the application of various assays. Very recently, Fierascu et al. (2018) quantified antioxidant potential of burdock extracts using DPPH (2,2-diphenyl-1-picrylhydrazyl) and phosphomolybdate assays to demonstrate that burdock extracts have very high antioxidative activities, presumably due to the high content of polyphenols (Fierascu et al., 2018). The potent antioxidative property makes these extracts effective inhibitors of lipid peroxidation in rat liver homogenate *in vitro* (Duh, 1998) and an excellent hepatoprotective agent *in vivo* and *in vitro* (Lin et al., 2000). Due to its radical scavenging ability, *A. lappa* is also used to treat gastrointestinal ulcers (da Silva et al., 2013).

### Antimicrobial Effects

Extracts of different parts of *A. lappa* have been investigated for their microbial-modulatory properties by many researchers. An organic extract from *A. lappa* has shown inhibiting properties toward the growth of *Pseudomonas aeruginosa, Escherichia coli, Lactobacillus acidophilus, Streptococcus mutans*, and *Candida albicans* residing in the teeth of the oral cavity (Gentil et al., 2006). Pereira et al. (2005) further reported potent growth inhibiting activities of *A. lappa* extract against a broad spectrum of oral microorganisms, specifically those associated with teeth infections, namely *Enterococcus faecalis, Staphylococcus aureus, Pseudomonas aeruginosa, Bacillus subtilis*, and *Candida albicans* (Pereira et al., 2005). Very recently, Fierascu et al. (2018) investigated antifungal potential of hydroalcoholic extract of burdock roots and observed that it is active against the fungal lines *Aspergillus niger* ATCC 15475 and *Penicillium hirsutum* ATCC 52323 (Fierascu et al., 2018). Fruit extract of *A. lappa* was tested (Dias et al., 2017) for *in vitro* antiviral properties against *Herpes simplex* virus type-1 (HSV-1) and was found to decrease viral load significantly at all concentrations tested (400, 50, and 3.125 μg/mL). At 400 μg/mL concentration, it showed comparable antiviral activity as acyclovir (50 μg/mL). Arctigenin, one of the key constituents of *A. lappa* extract, has shown potent activities against human immunodeficiency virus type-1 (HIV-1) both *in vivo* and *in vitro* presumably by increasing the expression of Heme oxygenase-2 (HO-2) and blocking HIV-1gag proteins (Schroder et al., 1990).

### Anti-inflammatory Effects

Various parts of *A. lappa* demonstrated anti-inflammatory effects (Liu et al., 2005). Burdock extract is known to alleviate wound irritation and swelling and therefore has been traditionally used for healing burn wounds. This effect might be mediated through the inhibition of the cyclooxygenase-2 (COX-2) enzyme. Cyclooxygenase is a lipid metabolizing enzyme that catalyzes the oxygenation of polyunsaturated fatty acids. This process forms prostanoids, specifically eicosanoids, which are known to be potent cell signaling molecules connected to inflammatory processes (Charlier and Michaux, 2003). Phenolic compounds present in burdock extract (e.g., arctigenin, lappaol F, diarctigenin) are inhibitors of this enzyme (Zhao et al., 2009; Lee and Kim, 2010), thereby suppressing lipopolysaccharide (LPS)-stimulated NO production (Park et al., 2007) and pro-inflammatory cytokines secretion (including TNF-α and IL-6) in a dose-dependent manner (Zhao et al., 2009; Kwon et al., 2016). Arctigenin also strongly inhibited the expression of iNOS (Inducible Nitric Oxide Synthase) and its enzymatic activity (Wang et al., 2007; Zhao et al., 2009). Moreover, it induced endothelial nitric oxide synthase (eNOS) and supressed in a rat model subarachnoid hemorrhage-induced vasospasm by regulation of the PI3K/Akt signaling pathway (Chang et al., 2015). Among the studied phenolic compounds, diarctigenin was found to inhibit the DNA binding ability of NF-κB and to inhibit NF-κB-regulated iNOS expression (Kim et al., 2008), thereby overall targeting NF-κB-activating signaling cascade directly to confer anti-inflammatory response. Luteolin, an important flavonoid from burdock was also reported to possess significant anti-inflammatory properties (Ferracane et al., 2010; Nabavi et al., 2015).

### Effects Against Skin Conditions

Leaves of *Arctium* species have been used in traditional medicinal practices in various skin conditions (e.g., rashes, boils, eczema, ichthyosis, acne, psoriasis, and abscesses) presumably due to the presence of various phenolic compounds. The potent antioxidant and anti-inflammatory properties of these compounds serve to detoxify and mediate healing action (Chan et al., 2011). Several hydroxycinnamic acids which are among the active phytochemicals in the *A. lappa* extracts (Liu et al., 2012; Tousch et al., 2014) have been found to act as free radical scavengers and possess antioxidant activities, which confer them potential to serve as skin protectors and wound healers (Graf, 1992; Phan et al., 2001; Taofiq et al., 2017). In addition, hydroxycinnamic acid derivatives also display anti-collagenase, anti-inflammatory, antimicrobial and anti-tyrosinase activities, as well as ultraviolet (UV) protective effects, suggesting that they can be exploited as anti-aging and anti-inflammatory agents, preservatives and hyperpigmentation-correcting ingredients (Ahangarpour et al., 2017). These bioactivities are the reason why burdock extracts find their use in various commercial cosmetic products.

### Effect on Potency and Fertility

Diabetes mellitus induces many complications among which dysfunctions male reproductive system is worth mentioning. Glucose metabolism plays an important regulatory role on the production or development of mature spermatozoa (spermatogenesis) as well as on maintaining specific functions, such as motility and fertilization ability in mature sperm cells. Therefore, it is not surprising that *A. lappa* root extract, which has hypoglycemic and antioxidative properties, would have beneficial effects on male potency and fertility. Ahangarpour et al. (2015) investigated the effect of *A. lappa* root extract on gonadotropin, testosterone, and sperm parameters in nicotinamide/streptozotocin-induced diabetic mice (Ahangarpour et al., 2015). The root extract led to increased level of luteinizing hormone (LH), follicle stimulating hormone (FSH), and testosterone as well as enhancement in sperm viability only in diabetic mice compared with the control group, indicating *A. lappa* root extract to be a potentially effective treatment for male sterility arising from diabetic conditions.

### Effect on NO Production

It was reported that arctigenin inhibited NO release by IFN-γ signal, whereas it significantly enhanced lipopolysaccharide-triggered NO production in RAW264.7 cells, suggesting that arctigenin may regulate immune responses in activated macrophages and lymphocytes including TNF-α and NO production and lymphocyte proliferation (Cho et al., 1999). Another study shows that arctigenin suppressed the overproduction of NO through down-regulation of iNOS expression and iNOS enzymatic activity in LPS-stimulated macrophage (Zhao et al., 2009). Besides, lappaol F and diarctigenin from *Arctium lappa* were shown to significantly inhibit NO production in the LPS-stimulated RAW264.7 cells with IC_50_ values of 9.5 and 9.6 μM, respectively (Park et al., 2007).

### Safety Considerations on *Arctium* Species

Several adverse effects have been reported in literature stemming from long-term use of *A. lappa*. For example, contact dermatitis might develop after several days of applying a burdock root plaster to a wound, or even as fast as within 12 h in some cases (Rodriguez et al., 1995). In one instance, anticholinergic poisoning has been reported upon oral consumption of *A. lappa* extract (Force, 2001). However, this poisoning later turned out to be caused by products that have been contaminated with root of belladonna (deadly nightshade). The latter herb contains the poisonous chemical atropine. Long-term consumption of burdock also has led to anaphylaxis in one case (Chan et al., 2011). Root oil made from *A. lappa* was also found to cause unfavorable physiological effects such as redness, and anaphylactic shock (Rodriguez et al., 1995; Lewis and Elvin-Lewis, 2003; Sasaki et al., 2003). Caution is advised for pregnant or nursing women to consume burdock or its extract, as it might have detrimental effects on the fetus (Chan et al., 2011). Burdock can also interfere with blood clotting. People who are already on blood thinning medications are advised not take it without approval from their doctors. Even though burdock is considered a ‘safe’ food, consuming it in large amounts should be avoided due to lack of large amount of safety studies on burdock. More *in vivo* studies are in particular needed on *A. lappa* to further evaluate its therapeutic potential and safe application window. Due to the presence of sesquiterpene lactones, the use of *Arctium* species should be avoided in patients with hypersensitivity to Asteraceae/Compositae (Chan et al., 2011).

## Summary

In summary, the volatile and non-volatile secondary metabolites present in different parts of *Arctium* species showed pharmacological potential in the treatment of various diseases. The literature existing on extracts of different parts of *A. lappa* and isolated compounds demonstrates antipyretic, antimicrobial, diuretic, diaphoretic, hypoglycaemic, antioxidant, anti-inflammatory, anti-hepatotoxicity, antiulcer, antimutagenicity, and antitumour activities. Hence, *Arctium* species display a broad therapeutic potential but further studies are needed on potential risks associated with their application.

## Author Contributions

All authors listed have made a substantial, direct and intellectual contribution to the work, and approved it for publication.

## Conflict of Interest Statement

The authors declare that the research was conducted in the absence of any commercial or financial relationships that could be construed as a potential conflict of interest.

## Footnotes

1 http://www.theplantlist.org
